# Polymorphisms and Pharmacogenomics for the Clinical Efficacy of Methotrexate in Patients with Rheumatoid Arthritis: A Systematic Review and Meta-analysis

**DOI:** 10.1038/srep44015

**Published:** 2017-03-07

**Authors:** Qi Qiu, Jing Huang, Xiaoming Shu, Huizheng Fan, Youwen Zhou, Cheng Xiao

**Affiliations:** 1 Institute of Clinical Pharmacology, Beijing Anzhen Hospital, Capital Medical University, Beijing 100029, China; 2 Institute of Clinical Medicine, China-Japan Friendship Hospital, Beijing 100029, China; 3 Beijing University of Chinese Medicine, Beijing 100029, China; 4 Department of Rheumatology, China-Japan Friendship Hospital, Beijing 100029, China; 5 Department of Gastroenterology, People’s Hospital of Yichun, Jiangxi Yichun 336000, China; 6 Department of Dermatology and Skin Science, University of British Columbia, Vancouver, BC, Canada; 7 Molecular Medicine Lab and Chieng Genomics Center, Vancouver Coastal Health Research Institute, Vancouver, Canada

## Abstract

Methotrexate (MTX) is widely used and considered a first-line disease modifying anti-rheumatic drug (DMARD) for the treatment of rheumatoid arthritis (RA). Many of the relevant genes have been investigated to estimate the association between gene polymorphisms and MTX effectiveness in RA patients, although inconsistent results have been reported. A systematic review and meta-analysis were performed to identify genetic variants associated with MTX efficacy. A total of 30 publications that included 34 genes and 125 SNPs associated with the transporters, enzymes, and metabolites of MTX or the progression of RA were included in the systematic review (SR), and 21 studies were included in 9 meta-analyses. Associations between MTX response in RA patients in MTHFR 1298A > C (rs1801131), ATIC 347C > G (rs2372536), RFC-1 80G > A (rs1051266), SLC19A1 A > G (rs2838956) and SLC19A1 G > A (rs7499) genetic polymorphisms were found, but not observed between the MTHFR 677C > T (rs1801133), TYMS 28 bp VNTR (rs34743033), MTRR 66A > G (rs1801394), and ABCB1 3435C > T (rs1045642). However, for the polymorphisms not being associated following meta-analysis could still be associated if larger cohorts were used, and studies of other polymorphisms are necessary in large cohorts and a rigorous way, which may provide more accurate results for the effect of the gene polymorphisms on the MTX response.

Rheumatoid arthritis (RA) is a systemic autoimmune disease characterized by chronic synovial joint inflammation, which leads to disability and diminished quality of life^1^,^2^. The main objectives for managing RA are to control pain, prevent or control joint damage and avoid long-term loss of function. Disease modifying anti-rheumatic drugs (DMARDs) are mainstay treatments for controlling the symptoms of RA and modifying its radiographic progression^3^. There are several DMARDs available; however, since the re-introduction of methotrexate (MTX) in the early 1980 s, MTX has become the most highly effective, fast-acting, disease modifying anti-rheumatic drug and is one of the most widely used and the first-line DMARD for the treatment of RA^4^,^5^. Accumulating evidence has indicated that earlier treatment with DMARD therapy improves long-term outcomes; therefore, identifying stable and reliable predictors of the MTX response is important for RA treatment^3^,^6^,^7^.

Although the combined efficacy and continuation rates for MTX are superior to that of other DMARDs^3^, considerable interpatient and intrapatient variability has been observed. Estimates indicate that up to one-third of patients fail to respond to treatment because of a lack of efficacy, and this variation in response limits the treatment options for certain patients^8^,^9^. Various factors, including individual patient factors, disease-specific factors and genetic factors, have been shown to influence treatment response^10^. Therefore, consistently reliable clinical or molecular markers are not available to accurately predict the response to MTX therapy. Pharmacogenomics refers to the study of the entire genome (covering transcriptomic and proteomic fields) and the expression levels of individual genes (mRNA) to identify the genetic factors influencing RA responses to MTX treatment^11^. Researchers believe that pharmacogenetic markers may offer a strategy to help identify patients who are more likely to respond to MTX, although this hypothesis requires clinical evidence.

The actual mechanisms of action of low-dose MTX in treating RA are not fully understood. Many of the relevant genes involved in the metabolism of MTX and progression of RA have been investigated to estimate the association between gene polymorphisms and MTX effectiveness in RA patients. However, these studies have produced mixed results because of their small sample size and poor statistical power. A meta-analysis can provide a potential solution to this problem because these evaluations combine the results from several studies^4^. Indeed, one of the major advantages of using meta-analyses is the ability to evaluate larger sample sizes, which reduces the likelihood of random errors producing false-positive or false-negative associations. Therefore, to overcome the limitations of individual studies, resolve inconsistencies, and increase precision, we performed a meta-analysis in our study to determine whether the gene polymorphisms in the evaluated studies can predict non-responsiveness to MTX therapy in patients with RA.

Over the past 10 years, seven meta-analyses^1^,^2^,^3^,^5^,^12^,^13^,^14^ on the association between polymorphisms and the clinical efficacy of MTX in RA patients were published in the PubMed and Embase databases. To the best of our knowledge, this is the first systematic review (SR) summarizing all of the available studies on the association between SNPs or VNTR polymorphisms and responsiveness to MTX in RA patients. In the present study, we focused on studies that reported the effects of MTX monotherapy and utilized pharmacogenetics, or the analysis of an individual’s genetic variations to predict RA responses to MTX treatment^11^. We updated the meta-analysis of the MTHFR (677C > T (rs1801133) and 1298A > C (rs1801131)), ABCB1 3435C > T (rs1045642), RFC-1 80G > A (rs1051266), and ATIC 347C > G (rs2372536) polymorphisms, completed the first meta-analysis on the association between SLC19A1 G > A (rs7499), SLC19A1 A > G (rs2838956), TYMS 28 bp VNTR (rs34743033) and MTRR 66A > G (rs1801394) polymorphisms and the effectiveness of MTX in RA patients. The MTHFR 677C > T (rs1801133), MTHFR 1298A > C (rs1801131), RFC-1 80G > A (rs1051266) and ATIC 347C > G (rs2372536) polymorphisms were included in the homology subgroup analysis.

## Results

### Study selection

**Figure 1:**
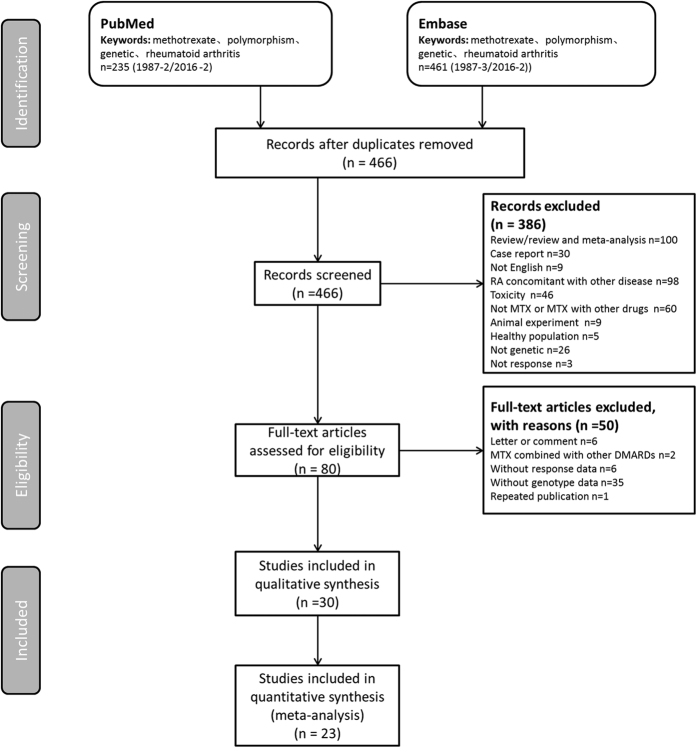
Study selection flow diagram adapted from the Preferred Reporting Items for SRs and Meta-Analyses (PRISMA) Statement.

Figure 1 shows the study selection process. The initial search identified 696 publications (PubMed: 235; and Embase: 461). The full text of 80 articles was reviewed in detail, and 50 articles were further excluded for the following reasons: letter or comment (n = 6), MTX combined with other DMARDs (n = 2), no response data (n = 6), no genotype data (n = 35), and repeated publication (n = 1). Ultimately, 30 publications were included in the SR and 21 studies were included in 9 meta-analyses.

### Study characteristics

**Figure 2:**
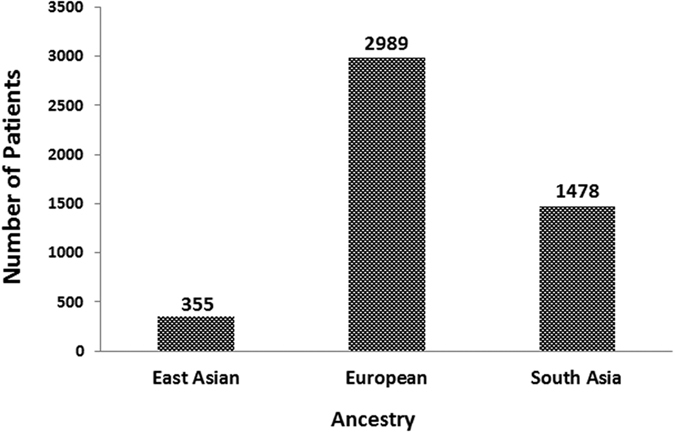
Distribution of ancestry in 28 studies that measured the association between polymorphisms and the response to MTX in RA.

For the analyzed studies, the characteristics and detected genes are shown in Table 1. The number of patients from Europe and South Asia accounts for a large population of the total number of publications (Fig. 2). All of the included studies were published in the last ten years.

### Pharmacogenetic markers of RA response to MTX treatment

A total of 34 genes with 125 gene SNPs associated with the transporters, enzymes, and metabolites of MTX or the progression of RA were evaluated to explore the association between the gene polymorphisms and the patient responses to MTX in previous studies (Table 1).

The main action of MTX is to inhibit the folate pathway and exert antiproliferative and anti-inflammatory effects in RA. An analysis of the MTX metabolic pathway showed that MTX enters target cells through reduced folate carriers (SLC16A7, SLC19A1 (RFC-1), SLC46A1, SLC22A11 and SLCO 1B1) and effluxes from target cells through ATP-binding cassettes (ABCs), predominantly ABCC1-4, ABCB1 and ABCG2. MTX is polyglutamated by the enzyme FPGS, and this type of polyglutamation can be reversed by the enzyme GGH. Polyglutamated MTX (MTX-PG) is retained within the cells and can competitively inhibit the activity of DHFR and reduce dihydrofolate to tetrahydrofolate (THF). THF is the precursor of the biologically active folate cofactor 5-methyl-THF, and this conversion is catalyzed by MTHFR. MTHFR, SHMT and other enzymes in one carbon pool (MS and MTRR) are not directly inhibited by MTX, although their expression level may contribute to the antifolate effects of MTX through subtle alterations in the folate pools^9^,^15^. MTX-PG can inhibit the TYMS (TSER)-mediated conversion of deoxyuridylate to deoxythymidylate in the de novo pyrimidine biosynthetic pathway and can also inhibit the activity of the enzyme ATIC and promote the intracellular accumulation of adenosine (AICAR), which through a series of enzymatic reactions, leads to the generation of adenosine and increased extracellular concentrations of adenosine, an anti-inflammatory agent. This pathway includes the intermediates inosine monophosphate and inosine triphosphate and the key enzymes ITPA, IMP (IMPDH) and AMP (AMPD1 and ADA). CCND1 controls cell progression through the G1/S phase and is also involved in the regulation of TYMS (TSER) and DHFR.

Gene polymorphisms associated with RA progression, including AIF-1, ESR a (ESR1) and ESR b (ESR2), PTPN22, HLA-DRB1 and HLA-DQB1, TGFB1, TLR4, CXCL9 and CXCL10, were also included in the pharmacogenetic marker studies of MTX response in RA patients.

**Figure 3:**
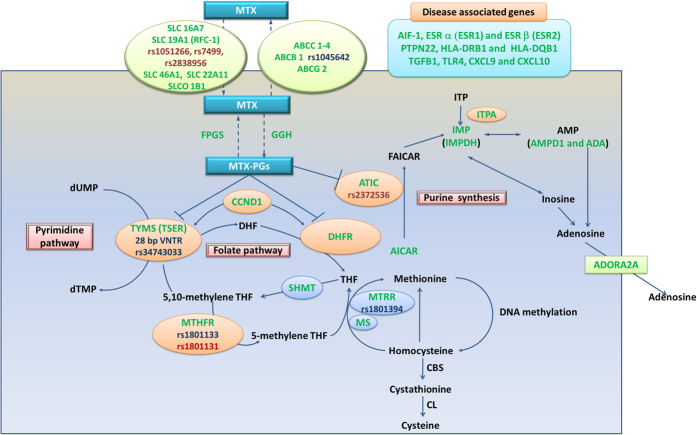
Summary of detected gene polymorphisms associated with the MTX response in RA patients in previous studies. Schematic representation of the intracellular folate biosynthetic pathway and the gene polymorphisms detected in previous studies (in green), the polymorphisms included in the present meta-analysis were highlighted in red or blue, and the SNPs in red showed associations with the MTX effectiveness in RA patients. MTX: Methotrexate; MTX-PGs: Methotrexate polyglutamates; Transport system: SLC16A7, SLC19A1, SLC46A1 and SLC22A11: Solute carriers; SLCO 1B1: Solute carrier organic anion transporter; RFC-1: Reduced folate carrier 1; ABCC1–4, ABCB1 and ABCG2: Adenosine triphosphate–binding cassette (ABC) transporters. Enzymes: ADA: Adenosine deaminase; ATIC: -Aminoimidazole-4-carboxamide ribonucleotide transformylase/IMP cyclohydrolase; IMP: Inosine monophosphate; IMPDH2: Inosine 5′-monophosphate dehydrogenase; CBS: Cystathionine-β-synthase; CL: Cystathionine lyase; DHFR: Dihydrofolate reductase; FPGS: Folylpolyglutamyl synthase; GGH: Glutamyl hydrolase; CCND1: Cyclin D1; MS: Methionine synthase; MTHFR: Methylenetetrahydrofolate reductase; MTHFD1: Methylenetetrahydrofolate dehydrogenase; MTRR: Methionine synthase reductase; SHMT: Serine hydroxymethyltransferase; TYMS: thymidylate; TSER: Thymidylate synthase enhancer region. Metabolites: ADP: Adenosine diphosphate; AICAR: 5-aminoimidazole-4-carboxamide ribonucleotide; AMP: Adenosine monophosphate; ATP: Adenosine triphosphate; CH3: Methyl group; DHF: Dihydrofolate; dTMP: Deoxythymidine-5′-monophosphate; dUMP: Deoxyuridine-5′-monophosphate; FAICAR: 10-formyl-AICAR; ITP: Inosine triphosphate; ITPA: Inosine triphosphate pyrophosphatase; THF: tetrahydrofolate. Disease associated gene polymorphisms: ESR: Estrogen receptors; AIF-1: Allograft inflammatory factor-1; PTPN2: Protein tyrosine phosphatase non-receptor type 22; HLA: Human leukocyte antigen; TLR4: Toll-like receptor 4; CXC: Chemokines; TGFB: tissue growth factor β.

The aforementioned genes are commonly used as important candidate gene polymorphisms in studies of RA response to MTX treatment. All of the genes and pathways included in the present SR are summarized in Fig. 3, where they were highlighted in green; the polymorphisms included in the present meta-analysis were highlighted in red or blue, and the SNPs in red showed associations with the MTX effectiveness in RA patients.

### MTHFR 677C > T (rs1801133)

Ten studies were included in the meta-analysis of MTHFR 677C > T (rs1801133), which contained data from a combined total of 579 responders and 677 nonresponders and included six European studies (423 responders and 438 nonresponders) and three South Asian studies (94 responders and 208 nonresponders). The characteristics of these studies are described in Table 2.

When all of the samples were included, the association between the frequency of 3 MTHFR 677C five > T (rs1801133) alleles (CC, CT and TT) and MTX response was not significant in pre allele (OR = 0.969, 95% CI: 0.768–1.222, Z = 0.26, P = 0.792), dominant (OR = 0.937, 95% CI: 0.734–1.197, Z = 0.52, P = 0.604), recessive (OR = 0.851, 95% CI: 0.564–1.285, Z = 0.77, P = 0.444), codominant (OR = 1.128, 95% CI: 0.884–1.439, Z = 0.97, P = 0.332), and homozygotic model (OR = 1.092, 95% CI: 0.703–1.696, Z = 0.39, P = 0.696). Moreover, significant between-study heterogeneity was not observed in all of the five models (Table 3).

Stratification by ethnicity did not identify a significant association between the MTHFR 677C > T (rs1801133) 3 allele frequency (CC, CT and TT) and MTX response in the European or South Asian populations in all of the five models (Table 3).

### MTHFR 1298A > C (rs1801131)

Eight studies were included in the meta-analysis of MTHFR 1298A > C (rs1801131), which contained data from a combined total of 425 responders and 527 nonresponders and included four European studies (270 responders and 288 nonresponders), two East Asian studies (79 responders and 48 nonresponders) and two South Asian studies (76 responders and 191 nonresponders). The characteristics of these studies are described in Table 4.

When all of the samples were included, the association between the MTHFR 1298A > C (rs1801131) 3 allele frequency (AA, AC and CC) and MTX response status was not significant in pre-allele model (OR = 1.004, 95% CI: 0.749–1.346, Z = 0.03, P = 0.979), dominant model (OR = 0.908, 95% CI: 0.596–1.382, Z = 0.45, P = 0.652), recessive model (OR = 0.861, 95% CI = 0.494–1.503, Z = 0.53, P = 0.599), codominant model (OR = 1.205, 95% CI = 0.772–1.882, Z = 0.82, P = 0.412), and homozygotic model (OR = 0.987, 95% CI = 0.626–1.554, Z = 0.06, P = 0.954). Moreover, significant between-study heterogeneity in codominant model (AC vs. AA + CC) was observed (I^2^ = 55.0%, χ^2^ = 15.54, P = 0.030) and East Asian subgroup in pre-allele model (I^2^ = 76.6%, χ^2^ = 4.28, P = 0.039) but not in other three models (Table 3).

**Figure 4:**
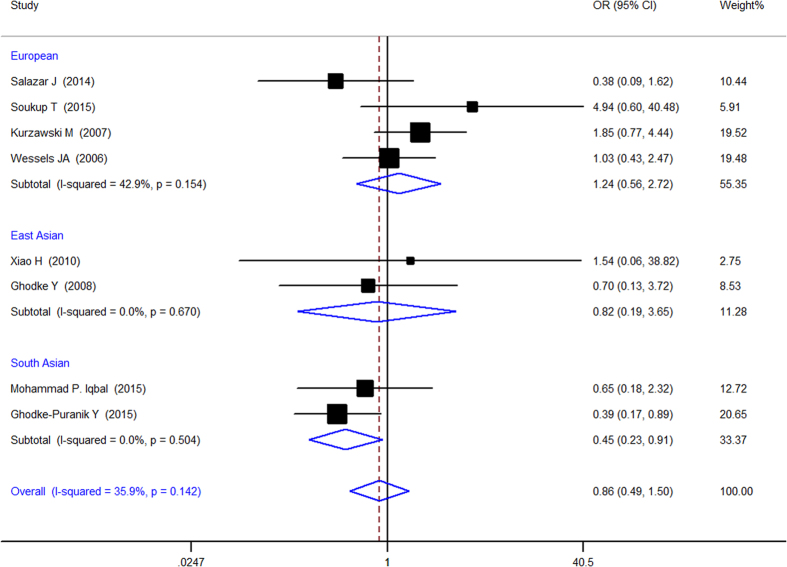
Forest plot showing the association between the MTHFR 677C > T (rs1801131) single-nucleotide polymorphism and the efficacy of methotrexate (CC vs. AC + AA (recessive Model)). % weight: the percentage weight attributed to each study in the meta-analysis; OR: odds ratio. Point estimates of the ORs for each study (black squares) and the corresponding 95% confidence intervals (CI) (horizontal lines) are shown, with the size of the black square representing the relative weight of the study. The diamonds represent the overall pooled estimate.

**Figure 5:**
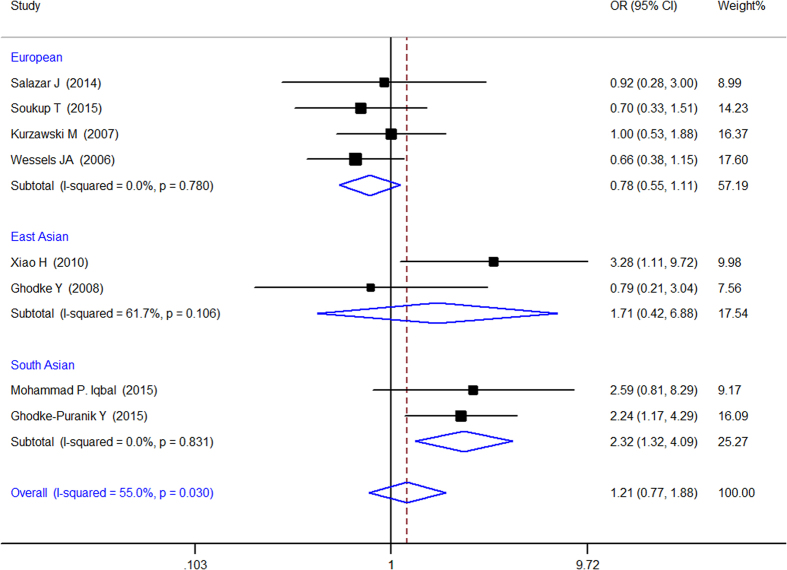
Forest plot showing the association between the MTHFR 1298A > C (rs1801131) single-nucleotide polymorphism and the efficacy of methotrexate (AC vs. AA + CC (codominant Model)). % weight: the percentage weight attributed to each study in the meta-analysis; OR: odds ratio. Point estimates of the ORs for each study (black squares) and the corresponding 95% confidence intervals (CI) (horizontal lines) are shown, with the size of the black square representing the relative weight of the study. The diamonds represent the overall pooled estimate.

Stratification by ethnicity identify a significant association between the MTHFR A1298C (rs1801131) 3 allele frequency (AA, AC and CC)and MTX response in the South Asian populations in recessive (OR = 0.454, 95% CI: 0.228–0.906, Z = 2.24, P = 0.025) (Fig. 4) and codominant model (OR = 2.319, 95% CI: 1.317–4.086, Z = 2.91, P = 0.004) (Fig. 5) but not in other models and other populations.

### ATIC 347C > G (rs2372536)

Five studies were included in the meta-analysis of ATIC 347C > G (rs2372536), which contained data from a combined total of 458 patients and 398 controls and included two European studies (132 responders and 134 nonresponders), one East Asian study (72 responders and 33 nonresponders) and two South Asian studies (254 responders and 231 nonresponders). The characteristics of these studies are described in Table 5.

**Figure 6:**
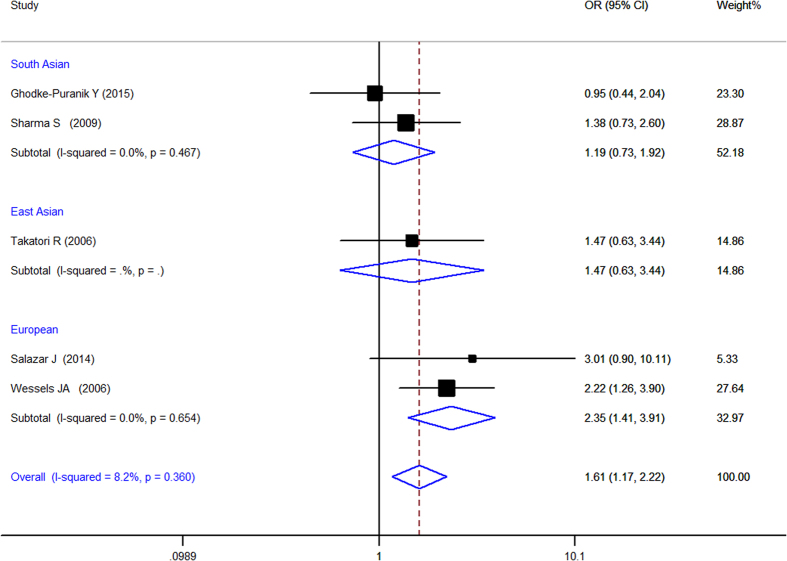
Forest plot showing the association between the ATIC 347C > G (rs2372536) single-nucleotide polymorphism and the efficacy of methotrexate (CC vs. CG + GG (dominant model)). % weight: the percentage weight attributed to each study in the meta-analysis; OR: odds ratio. Point estimates of the ORs for each study (black squares) and the corresponding 95% confidence intervals (CI) (horizontal lines) are shown, with the size of the black square representing the relative weight of study. The diamond represents the overall pooled estimate.

**Figure 7:**
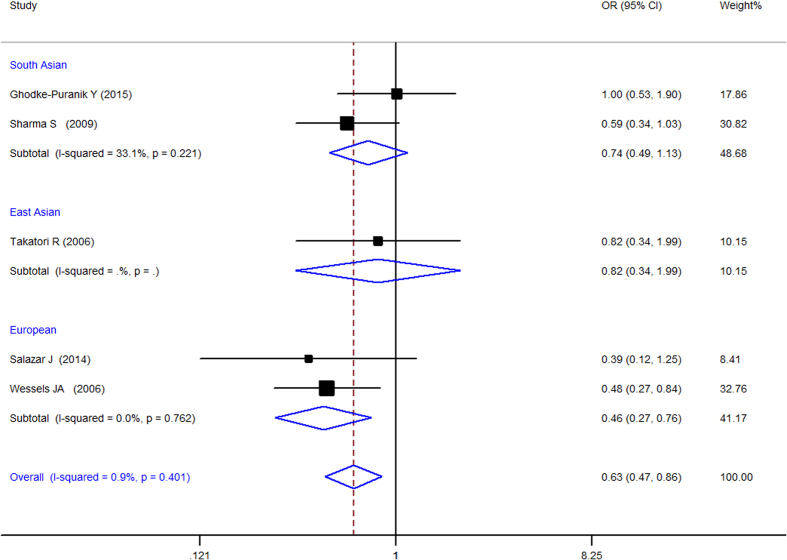
Forest plot showing the association between the ATIC 347C > G (rs2372536) single-nucleotide polymorphism and the efficacy of methotrexate (CG vs. CC + GG (codominant Model)). % weight: the percentage weight attributed to each study in the meta-analysis; OR: odds ratio. Point estimates of the ORs for each study (black squares) and the corresponding 95% confidence intervals (CI) (horizontal lines) are shown, with the size of the black square representing the relative weight of study. The diamond represents the overall pooled estimate.

When all of the samples were included, a significant association between the ATIC 347C > G (rs2372536) 3 allele frequency (CC, CG and GG) and MTX response status was identified in dominant model (OR = 1.612, 95% CI: 1.168–2.224, Z = 2.91, P = 0.004) (Fig. 6) and codominant (Fig. 7) model (OR = 0.634, 95% CI: 0.468–0.858, Z = 2.95, P = 0.003), but not in pre-allele model (OR = 1.263, 95% CI: 0.958–1.666, Z = 1.65, P = 0.098), recessive model (OR = 1.068, 95% CI: 0.699–1.630, Z = 0.30, P = 0.762) and homozygotic model (OR = 1.229, 95% CI: 0.749–2.015, Z = 0.82, P = 0.415). Moreover, significant between-study heterogeneity was not observed in all of the five models (Table 3).

Stratification by ethnicity identified a significant association between the ATIC 347C > G (rs2372536) 3 allele frequency (CC, CG and GG) and MTX response status in Europeans in pre-allele model(OR = 1.736, 95% CI 1.176–2.564, Z = 2.77, P = 0.006), dominant model (OR = 2.346, 95% CI 1.407–3.910, Z = 3.27, P = 0.001), and codominant model (OR = 0.458, 95% CI 0.274–0.764, Z = 2.99, P = 0.003) but not in other model and in the South Asian populations (Table 3).

### TYMS 28 bp VNTR (rs34743033)

Three studies were included in the meta-analysis of TYMS 28 bp VNTR (rs34743033), which contained data from a combined total of 335 responders and 264 nonresponders. The characteristics of these studies are described in Table 6.

When all of the samples were included, the association between the TYMS 28 bp VNTR (rs34743033) and MTX response status was not significant in pre-allele (OR = 1.174, 95% CI: 0.811–1.697, Z = 0.85, P = 0.396), dominant (OR = 1.238, 95% CI: 0.794–1.929, Z = 0.94, P = 0.347), recessive (OR = 0.787, 95% CI: 0.377–1.644, Z = 0.64, P = 0.524), codominant (OR = 1.093, 95% CI: 0.666–1.794, Z = 0.35, P = 0.724), and homozygotic model (OR = 1.400, 95% CI: 0.675–2.906, Z = 0.90, P = 0.366). Moreover, significant between-study heterogeneity was not observed in all of the five models (Table 3).

### MTRR 66A > G (rs1801394)

Two studies were included in the meta-analysis of MTRR 66A > G (rs1801394), which contained data from a combined total of 126 responders and 198 nonresponders. The characteristics of these studies are described in Table 7.

When all of the samples were included, the association between the MTRR 66A > G (rs1801394) allele frequency (AA, AG and GG) and MTX response status was not significant in pre-allele (OR = 1.088, 95% CI: 0.744–1.591, Z = 0.43, P = 0.664), dominant (OR = 1.165, 95% CI: 0.668–2.031, Z = 0.54, P = 0.590), recessive model (OR = 0.961, 95% CI = 0.502–1.841, Z = 0.12, P = 0.905), codominant model (OR = 0.897, 95% CI = 0.531–1.516, Z = 0.40, P = 0.686), and homozygotic model (OR = 1.188, 95% CI = 0.555–2.545, Z = 0.44, P = 0.657). Moreover, significant between-study heterogeneity was not observed in all of the five models (Table 3).

### RFC-1 80G > A (rs1051266)

Four studies were included in the meta-analysis of RFC-1 80G > A (rs1051266), which contained data from a combined total of 298 responders and 423 nonresponders. The characteristics of these studies are described in Table 8.

**Figure 8:**
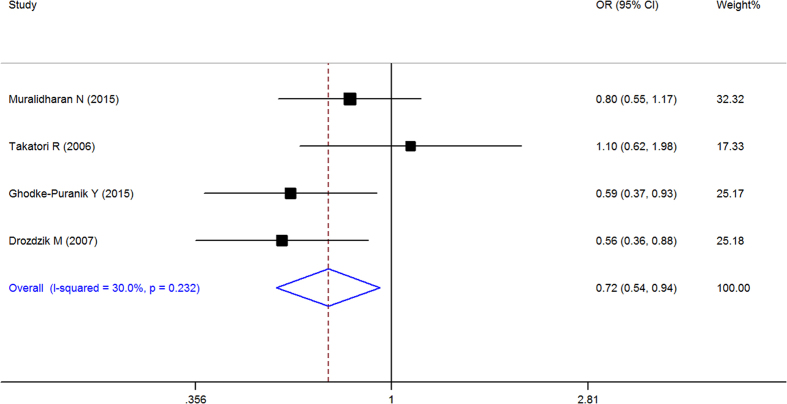
Forest plot showing the association between the RFC-1 80G > A (rs1051266) single-nucleotide polymorphism and the efficacy of methotrexate (G vs. A (Pre-allele model)). % weight: the percentage weight attributed to each study in the meta-analysis; OR: odds ratio. Point estimates of the ORs for each study (black squares) and the corresponding 95% confidence intervals (CI) (horizontal lines) are shown, with the size of the black square representing the relative weight of study. The diamond represents the overall pooled estimate.

**Figure 9:**
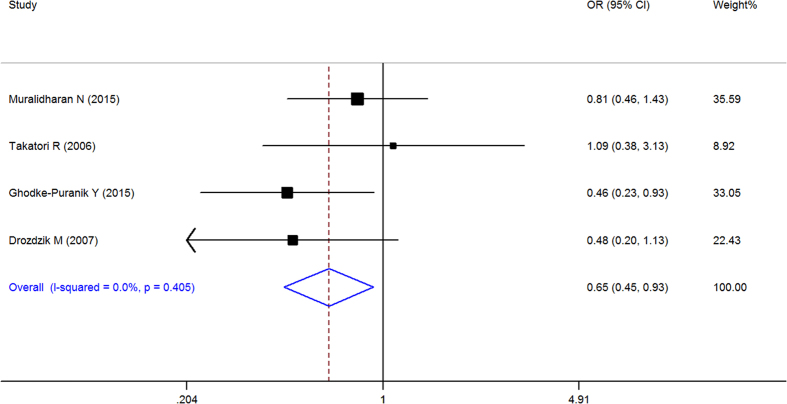
Forest plot showing the association between the RFC-1 80G > A (rs1051266) single-nucleotide polymorphism and the efficacy of methotrexate (GG vs. GA + AA (dominant model)). % weight: the percentage weight attributed to each study in the meta-analysis; OR: odds ratio. Point estimates of the ORs for each study (black squares) and the corresponding 95% confidence intervals (CI) (horizontal lines) are shown, with the size of the black square representing the relative weight of study. The diamond represents the overall pooled estimate.

**Figure 10:**
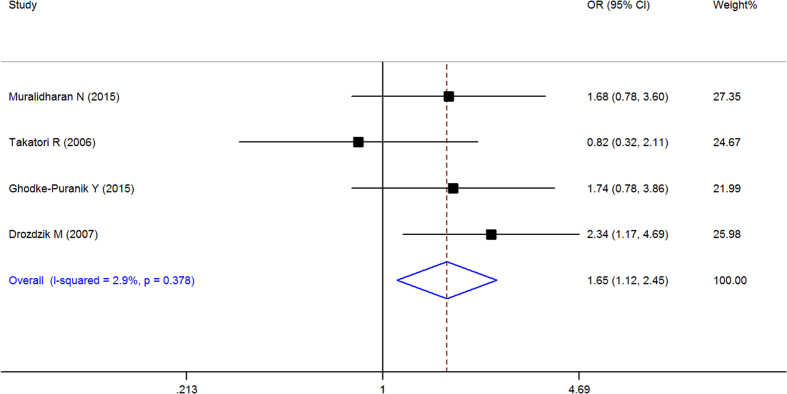
Forest plot showing the association between the RFC-1 80G > A (rs1051266) single-nucleotide polymorphism and the efficacy of methotrexate (AA vs. GA + GG (recessive Model)). % weight: the percentage weight attributed to each study in the meta-analysis; OR: odds ratio. Point estimates of the ORs for each study (black squares) and the corresponding 95% confidence intervals (CI) (horizontal lines) are shown, with the size of the black square representing the relative weight of study. The diamond represents the overall pooled estimate.

**Figure 11:**
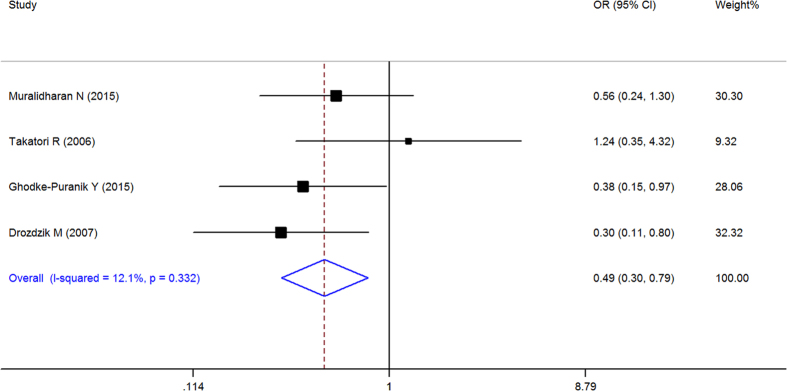
Forest plot showing the association between the RFC-1 80G > A (rs1051266) single-nucleotide polymorphism and the efficacy of methotrexate (GG vs. AA (homozygotic Model)). % weight: the percentage weight attributed to each study in the meta-analysis; OR: odds ratio. Point estimates of the ORs for each study (black squares) and the corresponding 95% confidence intervals (CI) (horizontal lines) are shown, with the size of the black square representing the relative weight of study. The diamond represents the overall pooled estimate.

When all of the samples were included, the association between the RFC-1 80G > A (rs1051266) allele frequency (GG, GA and AA) and MTX response status was significant in pre-allele (OR = 0.716, 95% CI 0.545–0.941, Z = 2.39, P = 0.017), dominant (OR = 0.645, 95% CI: 0.449–0.926, Z = 2.38, P = 0.017), recessive (OR = 1.653, 95% CI = 1.115–2.451, Z = 2.5, and P = 0.012), and homozygotic model (OR = 0.488, 95% CI = 0.302–0.789, Z = 2.93, P = 0.003), but not in codominant model (OR = 1.018, 95% CI = 0.743–1.396, Z = 0.11, P = 0.91). Moreover, significant between-study heterogeneity was not observed in all of the five models (Table 3, Figs 8–11).

Stratification by ethnicity identified a significant association between the RFC-1 80G > A (rs1051266) allele frequency (GG, GA and AA) and MTX response status in Europeans in pre-allele model(OR = 0.561, 95% CI 0.356–0.884, Z = 2.49, P = 0.013), recessive Model (OR = 2.343, 95% CI 1.169–4.694, Z = 2.40, P = 0.016), and homozygotic Model (OR = 0.301, 95% CI 0.114–0.796, Z = 2.42, P = 0.016), and South Asian populations in Pre-allele model (OR = 0.705, 95% CI 0.523–0.951, Z = 2.29, P = 0.022), dominant model (OR = 0.642, 95% CI 0.415–0.993, Z = 1.99, P = 0.046), and homozygotic model (OR = 0.473, 95% CI 0.252–0.887, Z = 2.33, P = 0.020), but not in other models (Table 3, Figs 8–11).

### SLC19A1 G > A (rs7499)

Two studies were included in the meta-analysis of SLC19A1 G > A (rs7499), which contained data from a combined total of 246 responders and 224 nonresponders. The characteristics of these studies are described in Table 9.

When all of the samples were included, the association between the SLC19A1 G > A (rs7499) 3 allele frequency (GG, GA and AA)and MTX response status was significant in pre-allele (OR = 1.536, 95% CI 1.176–2.005, Z = 3.15, P = 0.002), dominant (OR = 1.681, 95% CI: 1.146–2.467, Z = 2.66, P = 0.008), recessive (OR = 0.528, 95% CI = 0.316–0.884, Z = 2.43, P = 0.015), and homozygotic model (OR = 2.397, 95% CI = 1.359–4.229, Z = 3.02, P = 0.003), but not in the codominant model (OR = 0.861, 95% CI = 0.596–1.242, Z = 0.80, P = 0.423). Moreover, significant between-study heterogeneity was not observed in all of the five models (Table 3).

### SLC19A1 A > G (rs2838956)

Two studies were included in the meta-analysis of SLC19A1 A > G (rs2838956), which contained data from a combined total of 246 responders and 225 nonresponders. The characteristics of these studies are described in (Table 10).

When all of the samples were included, the association between the SLC19A1 A > G (rs2838956) 3 allele frequency (AA, AG and GG) and MTX response status was significant in pre-allele model(OR = 1.366, 95% CI 1.051–1.776, Z = 2.33, P = 0.020), recessive model (OR = 0.592, 95% CI = 0.361–0.969, Z = 2.08, P = 0.037) and homozygotic model (OR = 1.973, 95% CI = 1.131–3.443, Z = 2.39, P = 0.017), but not in the dominant (OR = 1.426, 95% CI = 0.965–2.109, Z = 1.78, P = 0.075) and the codominant model (OR = 0.98, 95% CI = 0.635–1.512, Z = 0.09, P = 0.927). Moreover, significant between-study heterogeneity was not observed in all of the five models (Table 3).

### ABCB1 3435C > T (rs1045642)

Two studies were included in the meta-analysis of ABCB1 3435C > T (rs1045642), which contained data from a combined total of 177 responders and 161 nonresponders. The characteristics of these studies are described in (Table 11).

When all of the samples were included, the association between the ABCB1 3435C > T (rs1045642) allele frequency and MTX response status was not significant in pre-allele (OR = 1.714, 95% CI 0.650−4.522, Z = 1.09, P = 0.276), dominant (OR = 1.755, 95% CI: 0.573–5.372, Z = 0.99, P = 0.325), recessive (OR = 0.429, 95% CI = 0.093–1.985, Z = 1.08, P = 0.279), codominant model (OR = 1.033, 95% CI = 0.666–1.602, Z = 0.15, P = 0.884), and homozygotic model (OR = 2.973, 95% CI = 0.401–22.016, Z = 1.07, P = 0.286). Moreover, significant between-study heterogeneity was observed in pre-allele (I^2^ = 86.9%, χ^2^ = 7.63, P = 0.006), dominant (I^2^ = 75.4%, χ^2^ = 4.07, P = 0.044), recessive (I^2^ = 83.4%, χ^2^ = 6.04, P = 0.014) and homozygotic model (I^2^ = 86.6%, χ^2^ = 7.48, P = 0.006) (Table 3).

### Publication bias

#### Publication bias for meta-analysis of MTHFR 677C > T (rs1801133)

**Figure 12:**
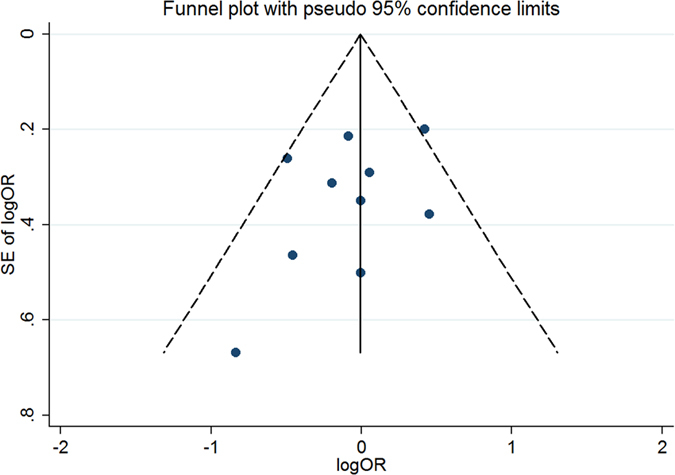
Funnel plot of studies that examined the association between the MTHFR 677C > T (rs1801133) polymorphism genotypes and MTX response.

The Begg’s funnel, presented in Fig. 12, did not indicate any evidence of publication bias. Neither Egger’s test (t = −1.23, P = 0.253) nor Begg’s test (Z = −0.98, P = 0.325) produced a statistically significant result.

#### Publication bias for meta-analysis of MTHFR 1298A > C (rs1801131)

**Figure 13:**
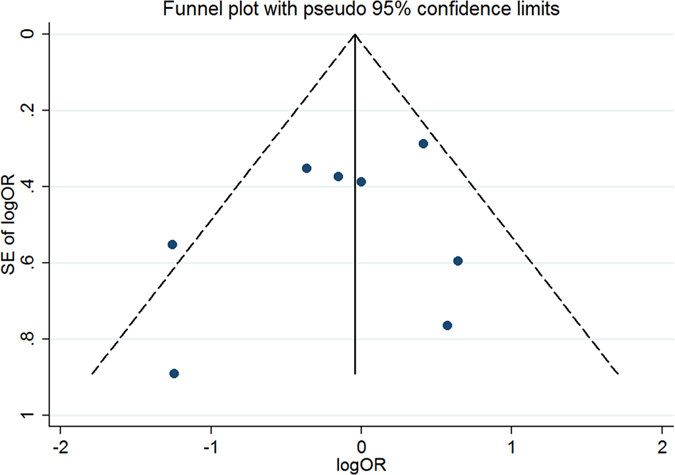
Funnel plot of studies that examined the association between the MTHFR 1298A > C (rs1801131) polymorphism genotypes and MTX response.

The Begg’s funnel, presented in Fig. 13, did not indicate any evidence of publication bias. Neither Egger’s test (t = −0.84, P = 0.433) nor Begg’s test (Z = −0.49, P = 0.621) produced a statistically significant result.

#### Publication bias for meta-analysis of ATIC 347C > G (rs2372536)

**Figure 14:**
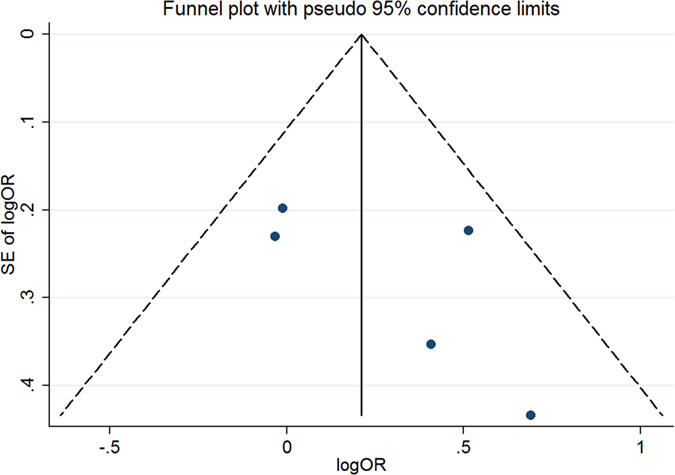
Funnel plot of studies that examined the association between the ATIC 347C > G (rs2372536) polymorphism genotypes and MTX response.

The Begg’s funnel, presented in Fig. 14, did not indicate any evidence of publication bias. Neither Egger’s test (t = 1.30, P = 0.286) nor Begg’s test (Z = 0.98, P = 0.327) produced a statistically significant result.

#### Publication bias for meta-analysis of RFC-1 80G > A (rs1051266)

**Figure 15:**
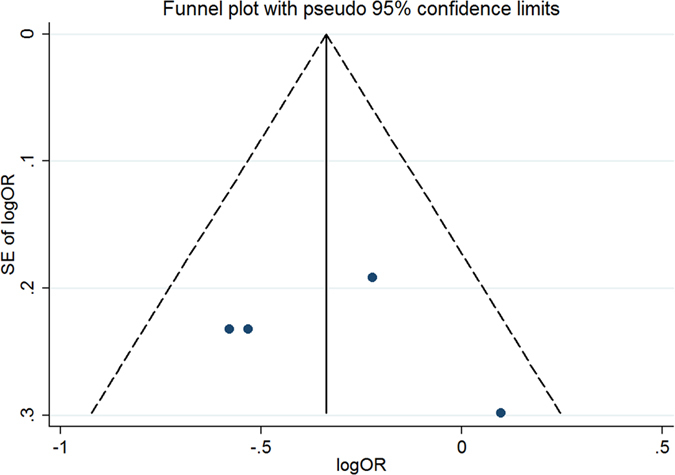
Funnel plot of studies that examined the association between the RFC-1 80G > A (rs1051266) polymorphism genotypes and MTX response.

The Begg’s funnel, presented in Fig. 15, did not indicate any evidence of publication bias. Neither Egger’s test (t = 0.47, P = 0.686) nor Begg’s test (Z = 0.68, P = 0.497) produced a statistically significant result.

## Discussion

The pathogenesis of RA is not well understood, and there are considerable challenges in the design of effective medicines to cure RA. MTX is still the gold standard drug for RA and plays antiproliferative and anti-inflammatory roles in RA therapy^11^,^16^. Although the factors influencing interpatient variability in MTX efficacy remain unclear, genetic factors related to drug metabolism and disease progression may play an important role in this variability. In recent years, extensive pharmacogenomics investigations have been performed to optimize MTX therapy for RA patients through genotyping and/or gene-expression-based tests. These tests were primarily based on mRNA and included transporters, enzymes, metabolites and disease associated genes^11^; however, the majority of the findings were inconclusive and inconsistent, even for classical candidate gene polymorphisms. Thus, developing effective and practical biomarkers to aid in the prediction of MTX responses in routine clinical practice remains a challenge. The present study performed an SR on the association between polymorphisms and the clinical efficacy of MTX in RA patients using papers published in the PubMed and Embase databases. Furthermore, this review focused on studies that reported the effects of MTX monotherapy and utilized pharmacogenetics, or the analysis of an individual’s genetic variation, to predict RA responses to MTX treatment.

Methylenetetrahydrofolate reductase (MTHFR) is the best studied gene in the MTX cellular pathway and encodes a protein with several important roles, including the conversion of the prominent circulatory form of folate, 5, 10-methylenetetrahydrofolate required for purine and thymidine synthesis, to 5-methyl tetrahydrofolate, which acts as a carbon donor for the re-methylation of homocysteine to methionine by methionone synthase^3^. MTHFR 677C > T (rs1801133) and 1298A > C (rs1801131) are the most well described two non-synonymous genetic variants, both of which have been reported to be associated with altered phenotypes. Patients with MTHFR 1298AA and MTHFR 677CC were reported to show a greater clinical improvement with MTX^17^. MTHFR 677C > T is a nonsynonymous polymorphism that results in the substitution of alanine with valine at codon 222 of the MTHFR enzyme^3^. It was reported that MTHFR 677TT carriers were statistically significant associated with more than 4-fold increased risk for nonresponse to MTX when compared to MTHFR677C carriers^18^. MTHFR 1298A > C is another nonsynonymous polymorphism that leads to the substitution of glutamine with alanine in the C-terminal regulatory domain of the MTHFR enzyme, which results in decreased enzyme activity^9^. In recent years, extensive investigations have been performed to identify the association between these two SNPs and MTX efficacy; however, the results were inconsistent. Ghodke-Puranik Y *et al*.^15^ reported that MTHFR 1298A allele (AA-AC) were more likely to have better MTX efficacy relative to those with MTHFR 1298 CC in Indian (South Asian) patients. However many other investigations did not shown an association between MTHFR 1298A > C allele and the MTX response in RA patients. The variability in individual study findings may arise due to the fact that each includes a small sample size thereby reducing the power to accurately estimate effect sizes. In the last decade, three meta-analyses were performed in relatively large samples, and the results suggested that both of the two SNPs were not associated with the efficacy of MTX in RA^3^,^12^,^13^. The present study updated the meta-analysis, and a significant association was not observed between either the 677C > T (rs1801133) allele (CC, CT and TT) or the 1298A > C (rs1801131) allele (AA, AC and CC) in all of the analyzed models when all of the samples were included. However, the significant association was founded in the South Asian populations in recessive (OR = 0.454, 95% CI: 0.228–0.906, Z = 2.24, P = 0.025)and codominant model (OR = 2.319, 95% CI: 1.317–4.086, Z = 2.91, P = 0.004) but not in other models and other populations. This is the first positive result in meta-analysis in MTHFR 1298A > C gene polymorphism in recent years.

ATIC is an important gene in the adenosine pathway, which is involved in the de novo synthesis of purine and converts aminoimidazole carboxamide adenosine ribonucleotide (AICAR) into formyl-AICAR and has been mapped to chromosome 2q35. MTX is polyglutamylated to form MTX polyglutamates after entering cells and directly inhibits ATIC, which leads to an intracellular accumulation of AICAR, and causing the release of adenosine into the extracellular space. The adenosine released diminishes the adherence of neutrophils and inhibits the function of natural killer cells, monocytes/macrophages and T-lymphocytes, thus producing potent anti-inflammatory effects^14^. ATIC 347C/G (rs2372536) polymorphism on exon 5 is the most commonly studied ATIC polymorphism in RA, and resulting in threonine to serine substitution at position 116 of the gene. Wessels JA *et al*.^19^ reported that individuals carrying the AMPD1 T allele and the ITPA 94CC and ATIC 347 CC genotypes were two to three times more likely to have a good clinical response to MTX. However, a lack of association has been reported between the ATIC 347C > G gene polymorphism and the MTX treatment response^15^,^20^,^21^,^22^,^23^. One meta-analysis found that the ATIC 347C > G polymorphism may be associated with non-responsiveness to MTX in Caucasian patients but not in Asian RA patients^14^. In the present meta-analysis, when all of the samples were included, a significant association between the ATIC 347C > G (rs2372536) 3 allele frequency (CC, CG and GG) and MTX response status was identified in dominant and codominant model but not in pre-allele (CC vs. CG + GG), recessive model (GG vs. CG + CC) and homozygotic (CC vs. GG) model. Further more, stratification by ethnicity identified the significant associations between the ATIC 347C > G (rs2372536) 3 allele frequency (CC, CG and GG) and MTX response status in Europeans in pre-allele, dominant and codominant model (CG vs. CC + GG). The results were consistent with the results of a previous study despite differences in the ethnicity classification method.

TYMS is a key enzyme in de novo thymidylate synthesis, and it is directly inhibited by MTX-PG. The TYMS gene has a tandem repeat polymorphism (two or three repeats of a 28 bp unit) in the enhancer region in the 5′-UTR. Lima A. *et al*.^24^ reported that the TYMS 28 bp VNTR (rs34743033) 3R3R polymorphism was associated with non-response to MTX. However, Wessels JA. *et al*.^19^ and Jekic B *et al*.^25^ did not find an association between this gene and the response to MTX. Three studies with a total of 603 European RA patients were included in the present meta-analysis^19^,^24^,^25^, and significant associations were not observed. Until now, Asian RA patients were not included in the research into this SNP.

MTR and MTRR participate in folate metabolism and are also involved in the metabolism of adenosine. MTRR is an auxiliary factor of MTR and catalyzes the regeneration of the methylcoamine, maintains sufficient activation of MTR, and is indirectly involved in the process of *in vivo* methylation. It was reported that the MTRR 66A > G gene polymorphism might affect the activity of the enzyme and the pharmacological effects of MTX, and MTR AG and MTRR G allele seems association with the poor response of MTX in RA patients^26^. Two studies were included in the present meta-analysis, including one for East Asian RA patients (n = 107) and another for South Asian RA patients (n = 217), and no significant association was observed between the MTRR 66A > G (rs1801394) genotype and MTX effectiveness.

Solute carriers (SLCs; especially SLC19A1/RFC-1) and ABCs (ABCC1-4, ABCB1 and ABCG2) are two groups of MTX transporters that influence cellular MTX uptake and efflux. The RFC-1 80G > A (rs1051266), SLC19A1 G > A (rs7499), SLC19A1 A > G (rs2838956), and ABCB1 3435C > T (rs1045642) polymorphisms were included in the present meta-analysis.

For RFC-1 80G > A (rs1051266), RFC-1 is a constitutively expressed folate transport protein that has high affinity for MTX and is involved in transport of folate and MTX into the cell; the 80G > A variant maps within exon 2 of the RFC1 gene on chromosome 21 and encodes a substitution of histidine for the arginine at amino acid position 27. Ghodke-Puranik Y *et al*.^15^ reported that those with an RFC1 80A allele (AA-GA) had better response to MTX than those with the RFC1 80 GG genotype. Drozdzik M *et al*.^27^ found that the patients with RFC-1 AA genotype responded to the therapy more effectively than carriers of AG and GG genotypes. Five studies with a total of 403 responders and 551 nonresponders were included in the present meta-analysis, and significant associations was observed between the allele frequency (GG, GA and AA) and MTX response status in pre-allele, dominant, recessive and homozygotic model, but not in codominant model when all of the samples were included. Moreover, stratification by ethnicity identified a significant association between the RFC-1 80G > A (rs1051266) allele frequency (GG, GA and AA) and MTX response status in Europeans in pre-allele, recessive and homozygotic model, and South Asian populations in pre-allele, dominant model and homozygotic model. This result was consistent with two previous meta-analyses, which found that the RFC-1 80G > A polymorphism is associated with responsiveness to MTX therapy^1^,^2^, even though the inclusion and exclusion criteria are different. In the present study, we only focused on the association between gene polymorphisms and the response to MTX monotherapy in RA patients and did not investigate toxicity^28^ and gene-gene interactions^29^. In addition, combined MTX and biologic disease-modifying anti-rheumatic drug (bDMARD) treatment^30^,^31^,^32^ studies and reviews^1^,^33^ were excluded from the meta-analysis of the RFC1 80G > A (rs1051266) polymorphism. Remarkably, the same SNP (rs1051266) was identified by a different name (SLC19A1 G > A) in the research from Lima A. *et al*.^34^, but was excluded in the present meta-analysis because it did not conforming to Hardy-Weinberg equilibrium.

The present meta-analysis of SLC19A1 A > G (rs2838956) found significant associations between the SLC19A1 A > G (rs2838956) 3 allele frequency (AA, AG and GG) and MTX response status in pre-allele recessive (A vs. G) and homozygotic model (AA vs. GG). For the SLC19A1 G > A (rs7499) gene polymorphism, this meta-analysis showed a significant association between the frequency of 3 alleles (GG, GA and AA) and MTX response status in in pre-allele, dominant, recessive and homozygotic model when all 480 patients were included in the present study. However, because of the small sample size, the association between SLC19A1 (rs2838956 and rs7499) and the response to MTX in RA patients require further verification.

Furthermore, the ABCB1 3435C > T (rs1045642) polymorphism, Takatori R *et al*.^35^ found that patients with ABCB1 3435CC and 3435CT showed higher therapeutic effects of MTX, which is inconsistent with the results of Lima A *et al*.^34^. When all of the samples were included in the present study, the association between the ABCB1 3435C > T (rs1045642) 3 allele frequency and MTX response status was not significant. This result is consistent with a previous meta-analysis that showed a negative association between the ABCB1 C3435T polymorphism and RA susceptibility or responsiveness to MTX^5^.

In addition to the above MTX transporter genes, an increased likelihood of non-response has been reported to be associated with SLC22A11 rs11231809 T carriers; ABCC1 rs246240 G carriers; ABCC1 rs3784864 G carriers; the CGG haplotype for ABCC1 rs35592, rs2074087 and rs3784864; and the CGG haplotype for ABCC1 rs35592, rs246240 and rs3784864^34^.

Many RA progression genes have been included in research investigating the association between gene polymorphisms and MTX response. SNPs in AIF-1^36^, ESR a (ESR1) and ESR b (ESR2)^37^, PTPN22^38^, HLA-DRB1 and HLA-DQB1^39^, TGFB1^40^, TLR4^40^, CXCL9 and CXCL10^41^ have been evaluated, although most of these studies showed a negative association between these polymorphisms and MTX effectiveness in RA patients with the exception of the AIF1 CC (rs2259571) genotype, which showed a poorer response to therapy with MTX^36^, and HLA-DRB1*03, which Ali AA *et al*.^39^ found to be significantly associated with nonresponders to MTX treatment, and suggested that Pakistani patients with this genotype are less likely to benefit from MTX.

The lack of a significant association in this meta-analysis may represent a true result, but the possibility of a false-negative finding requires consideration. Certain limitations of our meta-analysis warrant consideration. First, the possibility of publication bias is always a concern. Although our analysis did not observe clear evidence of such a bias, it should be recognized that publication bias is difficult to exclude with certainty, especially when the number of incorporated studies is small. Second, publication bias could have distorted our meta-analysis because of the small number of included studies. We included 10, 8, 5, 3, and 4 studies in the meta-analysis of the MTHFR (677C > T (rs1801133) and 1298A > C (rs1801131)), ATIC 347C > G (rs2372536), TYMS 28 bp VNTR (rs34743033), and RFC-1 80G > A (rs1051266) polymorphisms, respectively, and 2 studies in the meta-analysis of the MTRR 66A > G (rs1801394), SLC19A1 (G > A (rs7499) and A > G (rs2838956)), and ABCB1 3435C > T (rs1045642) polymorphisms. Third, heterogeneity and confounding factors may have affected the meta-analysis. Variables such as sex, rheumatoid factor status, disease duration, and even the baseline DAS28 all have the potential to influence this analysis.

Even though the genetic researches showed inconsisit results in the previous researches and meta-analysis, the genetics still seem to be a powerful supplemental method of the experssion and the biomarker studies in the future research into MTX response and the combination of the above research techniques should be helpful to understanding the MTX efficacy. Given the small effect size still a choke point of the polymorphisms associated research, genotyped these and other polymorphisms within the candidate genes in large sample size study are required. Furthermore, the ethnic group, sex, rheumatoid factor status, disease duration, MTX dose, treatment duration, MTX with or without the combination of the folic acid and even the baseline disease activity of the cohorts might greatly influence the correlation of genetic polymorphisms and the MTX efficacy, so the standardized research and treatment protocal is needed to improve the quality of the genetics researches.

Taken together, this SR and meta-analysis demonstrated associations between MTX response in RA patients in MTHFR 1298A > C (rs1801131), ATIC 347C > G (rs2372536), RFC-1 80G > A (rs1051266), SLC19A1 A > G (rs2838956) and SLC19A1 G > A (rs7499) genetic polymorphisms, but not in the MTHFR 677C > T (rs1801133), TYMS 28 bp VNTR (rs34743033), MTRR 66A > G (rs1801394), and ABCB1 3435C > T (rs1045642) genetic polymorphisms. However, for the polymorphisms not being associated following meta-analysis (e.g. those in MTHFR 677C > T (rs1801133) could still be associated if larger cohorts were used, and studies of other polymorphisms are necessary in large cohorts and a rigorous way, which may provide more accurate results for the effect of the gene polymorphisms on the MTX treatment response.

## Methods

The methodology for this study was based on the Preferred Reporting Items for SRs and Meta-Analyses (PRISMA) statement^42^. Ethical approval was not necessary for this meta-analysis because the results included pooled data from individual studies that received ethics approval.

### Published study identification and selection for meta-analysis

All studies investigating the relationship between a genetic variant and MTX treatment response in RA published before February 2016 were identified using computer-based searches of the PubMed database and Embase database (OvidSP) using the following combination of keywords: ‘methotrexate[Title/Abstract] AND (polymorphism[Title/Abstract] OR polymorphisms[Title/Abstract] OR genetic[Title/Abstract])) AND (“arthritis, rheumatoid”[MeSH Terms] OR (“arthritis”[All Fields] AND “rheumatoid”[All Fields]) OR “rheumatoid arthritis”[All Fields] OR (“rheumatoid”[All Fields] AND “arthritis”[All Fields]))’. Details of the search flow are provided in Fig. 1. The titles alone were initially reviewed for suitability, and then the abstracts of these titles were obtained and reviewed to determine the full-text retrieval suitability. Data were then extracted as described in the following section from suitable full-text reports.

### Inclusion and exclusion criteria

The following inclusion criteria have been used: (1) evaluation of the associations between the gene polymorphism (or nucleotide tandem repeat)and the efficacy of MTX treatment in adult patients with RA; (2) detailed genotype data could be acquired to calculate the odds ratios (ORs) and 95% confidence intervals (CIs); (3) per-reviewed publications in English or Chinese. Exclusion criteria include (1) duplication of previous publications; (2) comment, review, editorial and conference abstract; (3) inability to ascertain the number of null and wild genotypes or alleles; (4) studies not conforming to Hardy-Weinberg equilibrium; (5)studies with no SNP site or no gene sequence; (6)non-English or Chinese publications. Each study was screened in duplicate by two independent reviewers (QQ and HJ) per the guidelines of the Human Genomic Epidemiology (HuGE) Review Handbook. Of note, for studies of MTX efficacy, all measures of disease activity were accepted, which mainly included the 44-joint count Disease Activity Score (DAS44) or the 28-joint count DAS (DAS28) or Physician’s global assessment of disease (VAS score) and the ACR 20% or ACR 50% improvement response criteria (ACR20 or ACR50)^1^.

### Data extraction

References were screened and data were extracted independently by 2 authors (QQ and HJ) using a predetermined data collection template. To resolve discrepancies on the inclusion of studies and interpretation of data, a third investigator (XC) was consulted, and consensus was reached by discussion. The following data were recorded: first author’s last name, year of publication, location of study, inclusion and exclusion criteria, sample size, MTX dose, SNP analysis results, treatment duration, demographic details of patients, follow-up period, and outcomes.

### Statistical analyses

Hardy–Weinberg equilibrium (HWE) was accessed for each study by Chi-square test in response groups, and P < 0.05 was considered a significant departure from HWE. Studies that did not use a categorical outcome for response or did not publish necessary genotype counts per response category were excluded, if these data could not be obtained directly from the authors. The gene SNPs detected in more than two studies were included in the meta-analysis. Genotype frequencies for the MTHFR (677C > T (rs1801133) and 1298A > C (rs1801131)), ATIC 347C > G (rs2372536), TYMS 28 bp VNTR (rs34743033), MTRR 66A > G (rs1801394), RFC-1 80G > A (rs1051266), SLC19A1 (G > A (rs7499) and A > G (rs2838956)), and ABCB1 3435C > T (rs1045642) polymorphisms were determined. In this process pre-allele, dominant, recessive, codominant, and homozygotic model were performed and allowed for the inclusion of a maximum number of studies^12^,^43^,^44^. For each study, the point estimate of risk, the OR and the corresponding 95% CIs of MTX responders versus nonresponders were calculated. Then, the overall pooled OR and corresponding 95% CIs were estimated using the Mantel–Haenszel method, and the fixed effect was the absence of moderate inconsistency (>25%) across studies^3^. A fixed effect framework assumes that the effect of allele frequency is constant across studies and between-study variations are caused by chance or random variation. The random effects model was used when heterogeneity > = 25% and the fixed effect model was used when heterogeneity <25%, and it assumes different underlying effects, considers both within-and between-study variation and is advantageous because it accommodates diversity between studies and provides a more conservative estimate. The odds ratio (OR) was pooled using inverse variance methods to generate a summary OR and 95% confidence interval (CI). We assessed the heterogeneity between the included studies using the χ2-based Cochran’s Q statistic. The percentage of across-study variability attributable to heterogeneity beyond chance was estimated using the I^2^ statistic. Differences in the pooled ORs were compared using a Z test. Potential publication bias was assessed with the Egger’s test and represented graphically with Begg’s funnel plots of the natural log of the OR versus its standard error. A two-sided P value of less than 0.05 was considered significant for all analyses. All statistical meta-analyses were completed with STATA (version 13.0; Stata Corp, College Station, TX, USA)^45^.

## Additional Information

**How to cite this article**: Qiu, Q. *et al*. Polymorphisms and Pharmacogenomics for the Clinical Efficacy of Methotrexate in Patients with Rheumatoid Arthritis: A Systematic Review and Meta-analysis. *Sci. Rep.* **7**, 44015; doi: 10.1038/srep44015 (2017).

**Publisher's note:** Springer Nature remains neutral with regard to jurisdictional claims in published maps and institutional affiliations.

## Figures and Tables

**Table 1:** Studies reporting methods of associating polymorphisms with responses to MTX in RA.

Study	Number of patients	Patient Countries (Ancestry)	Evaluation of efficacy	Genotyping method	Genes	Individual study results
Lima A *et al*.^34^	233	Portugal (European)	DAS28 ≤3.2	Sequenom	SLC16A7 A > T (rs3763980), SLC16A7 T > G (rs10877333), SLC19A1 G > A (rs7499), SLC19A1 G > A (rs1051266), SLC19A1 A > G (rs2838956), SLC19A1 A > G, ABCC1 G > A (rs3784864)(rs2838956), SLC19A1 G > A (rs3788200), SLC22A11 T > A (rs11231809), SLC46A1 G > A (rs2239907), SLCO1B1 T > C (rs4149056), ABCB1 C > T (rs1045642), ABCB1 C > T (rs1128503), ABCB1 G > T(rs2032582), ABCC1 T > C (rs35592), ABCC1 A > G (rs246240), ABCC1 G > C (rs2074087), ABCC1 G > A (rs3784864), ABCC2 G > A (rs717620), ABCC2 C > T (rs4148396), ABCG2 T > C (rs13120400), ABCG2 G > A (rs17731538)	The results demonstrated that SLC22A11 rs11231809 T carriers were significantly associated with more than five-fold increased risk for non-response to MTX. Regarding SNPs in ABCs, ABCC1 rs246240 G carriers and ABCC1 rs3784864 G carrierswere associated with MTX non-response.
Muralidharan N *et al*.^27^	327	India (South Asia)	Improvement of >1.2 in DAS28 score and a DAS of ≤2.6 (remission) on follow-up	PCR-RFLP	RFC -1 80G > A (rs1051266)	RFC-1 80G > A genepolymorphism confers protection for RA. However, it is not associated with MTX treatment response and MTX-induced adverse effects in South Indian Tamil patients with RA.
Lima A *et al*.^24^	233	Portugal (European)	DAS28 ≤ 3.2	PCR-RFLP	TYMS 28 bp VNTR(rs34743033), TSER (rs2853542 and rs34743033), TYMS 1494del6 (rs34489327)	Considering TYMS genotypes, 3R3R, 3RC3RG and 6bp2 carriers were associated with non-response to MTX.
Salazar J *et al*.^23^	61 of 124	Spain (European)	DAS28 ≤ 3.2 and improvement from baseline was ≥1.2 or 3.2≤ DAS28 ≤ 5.1 together with an improvement between 0.6 and 1.2	Real-Time PCR	MTHFR (1p36.3) (rs13306561, rs9651118, rs11121832, rs4846052, rs17421511, rs1801133, rs1801131, rs1476413); DHFR (5q11.2-q13.2)(rs1650697, rs70991108, rs1643650); TYMS (18p11.32) (rs2847153, rs2847150, rs2847149, rs16948305); ATIC (2q35) (rs10197559, rs16853782, rs2372536, rs12995526, rs7586969, rs2177735, rs16853826); CCND1 (11q13)(rs9344, rs649392)	Two SNPs in the MTHFR gene, rs17421511 and rs1476413, and one in the DHFR gene, rs1643650, were significantly associated with response to MTX treatment in rheumatoid arthritis, We also found that two SNPs in the ATIC gene, rs16853826 and rs10197559, were associated with toxicity.
Lima A *et al*.^18^	233	Portugal (European)	DAS28 ≤ 3.2	PCR-RFLP and TaqMan	MTHFR 677C > T (rs1801133), ATIC 675T > C (rs4673993)	MTHFR 677TT carriers were statistically significant associated with more than 4-fold increased risk for nonresponse to MTX whencomparedtoMTHFR677Ccarriers. Additionally, ATIC 675T carrierswere statistically significant associated with more than 5-fold increased risk for nonresponse to MTX when compared to ATIC 675CC.
Pawlik A *et al*.^36^	221	Poland (European)	DAS28 ≤ 2.4 (patients with remission of disease symptoms)	TaqMan	AIF1 C > T (rs2269475), AIF1 G > A (rs2736182), AIF1 A > C (rs2259571)	The results of this study suggest that the patients with the rs2259571 CC AIF1 genotype have a poorer response to therapy with MTX.
Jekic B *et al*.^25^	184	Serbia (European)	DAS281 ≤ 3.2 and Δ DAS28 ≥ 1.2 or 3.2 < DAS281 ≤ 5.1 and 0.6 < ΔDAS28 ≤ 1.2	PCR-RFLP	GGH 452C > T, GGH -354 G > T, CCND1 870A > G, TYMS 2R/3R, TYMS 3RG/3RC	The 3G/3G genotype of the TYMS gene may indicate predisposition of poor response to MTX and GG genotype of GGH −354 T > G polymorphism may have high predictive value for myelosuppression in RA patients.
Owen SA *et al*.^46^	147 responders (309)	UK (European)	Physician statement of good response plus a stable dose of MTX for at least 6 months, with an ESR of 20 and/or normal CRP	Sequenom	ATIC rs7563206 C > T, ATIC rs3821353 G > T, ATIC rs12995526 C > T, ATIC rs16853834 C > T, GGH rs12681874 C > T, SLC19A1 rs11702425 T > C, SLC19A1 rs2838956 A > G, SLC19A1 rs7499 G > A, SLC19A1 rs2274808 C > T, SLC19A1 rs9977268 C > T, SLC19A1 rs7279445 C > T	Associations were detected with efficacyincluding four SNPs in the ATIC gene (rs12995526, rs3821353, rs7563206 and rs16853834), six SNPs in the SLC19A1 gene region (rs11702425, rs2838956, rs7499, rs2274808, rs9977268 and rs7279445) and a single SNP within the GGH gene (rs12681874), the results suggest that genetic variations in several key MTX pathway genes may influence response to MTX in the RA patients.
Milic V *et al*.^47^	125	Serbia (European)	DAS281 ≤ 3.2 and Δ DAS28 ≥ 1.2 or 3.2 < DAS281 ≤ 5.1 and 0.6 < DAS28 ≤ 1.2	PCR-RFLP	DHFR 216T > C (rs6151599), DHFR 317A > G (rs408626), ATIC 129T > G (rs4535042)	RA patients with DHFR-317AA genotype had less favourable response to MTX.
Majorczyk E *et al*.^38^	308	Poland (European)	ACR20	PCR-RFLP	PTPN22 1858C > T (rs2476601)	The response of RA patients to MTX treatment does not seem to depend on this SNP.
Xiao H *et al*.^48^	110	China (East Asian)	ACR20	TaqMan	MTHFR 677C > T (rs1801133), MTHFR 1298A > C (rs1801131), MTHFR G > A (rs2274976) and MTHFR C > T (rs2066462)	While rs1801131A/C genetic polymorphism is associated with the clinical response, rs1801133C/T and rs2274976A/G genetic polymorphisms are associated with MTX-related AEs in the treatment of RA.
Sharma S *et al*.^22^	273 of 281	India (South Asia)	DAS28-3＜3.2	PCR-REP	ATIC 347C > G (rs2372536), AMPD1 C > T (rs17602729), ADA C > G (rs1799880), ADA A > G (rs244076), ADORA2A T > C (rs5751876)	Genes from all the three pathways seem to contribute to MTX response in the Indian population.
Ghodke Y *et al*.^49^	34	India (South Asia)	ARC20	PCR-RFLP	MTHFR 677C > T (rs1801133), MTHFR 1298A > C (rs1801131), TS 5′UTR 2R/3R, TS 3′UTR − > +6 bp	Our findings do not suggest a significant association of MTHFR/TS allele/genotype with MTX response in our ethnically distinct Indian (Asian) RA patients.
Takatori R *et al*.^35^	124	Japan (East Asian)	Last maintenance dosage of MTX was ≤6 mg/week	Real-Time PCR	ABCB1 3435C > T (rs1045642), RFC-1 80G > A (rs1051266), ATIC 347C > G (rs2372536), TYMS 3UTR − > +6 bp	There were no significant differences in MTX sensitivity among the genotypes of RFC1, ATIC and TYMS genes, the results of this study indicated that patients with ABCB1 3435CC and 3435CT showed higher therapeutic effects of MTX.
Ali AA *et al*.^39^	91	Pakistan (South Asia)	50% or greater reduction in ESR, Richie index, number of swollen joints and morning stiffness compared with index at entry	PCR-SSP	HLA-DRB1 *01 *03 *04 *07 *08 *09 *10 *11 *12 *13 *14 *15 *16; HLA-DQB1 *02 *03 *04 *05 *06	RA susceptibility in most Pakistani patients is associated with the HLA-DRB1*01/DQB1*06 genotype. HLA-DRB1*03 was found to be significantly more common among non-responders to MTX treatment suggesting that Pakistani patients with this genotype are less likely to benefit from MTX
Mohammad Perwaiz Iqbal *et al*.^50^	67	Pakistan (South Asia)	50% or greater reduction in ESR, Richie index, number of swollen joints and morning stiffness compared with index at entry	PCR-RFLP	MTHFR 677C > T (rs1801133); MTHFR 1298A > C (rs1801131)	MTHFR C677T and A1298C polymorphisms are not associated with response to MTX in a population of Pakistani RA patients.
Kooloos WM *et al*.^40^	205	Netherlands (European)	DAS ≤ 2.4	Real-Time PCR	DHFR −829C > T (rs34764978); ABCB1 3435C > T (rs60023214); ITPA IVS2 + 21A > C (rs7270101); HLA-G − > +ATTTGTTCATGCCT (−14 bp > +14 bp) (rs16375); TGFB1 + 869T > C (rs1982073); TLR4 + 896A > G (rs4986790); IMPDH2 + 787C > T	No significant associations or replications of these genetic variants with MTX efficacy were demonstrated
Aggarwal P *et al*.^51^	150	India (South Asia)	DAS28＜3.2	PCR-RFLP	MTHFR 677C > T (rs1801133)	Our findings suggest that C677T polymorphism in the MTHFR gene is not predictive of toxicity or efficacy of MTX treatment in RA patients receiving folate supplementation.
Drozdzik M *et al*.^27^	174	Poland (European)	ACR20	PCR-RFLP	RFC-1 80G > A (rs1051266)	The patients with RFC-1 AA genotype responded to the therapy more effectively than carriers of AG and GG genotypes.
Shen S.-H *et al*.^26^	121	China (East Asian)	ACR20	Real-Time PCR	MTR2756A > G (rs1805087); MTRR 66A > G (rs1801394)	MTR AG and MTRR G allele seems association with the poor response of MTX in RA patients. The cumulative genotypes of MTR and MTRR may be used for the index to predict the good clinical response of patients who take MTX.
Muralidharan N *et al*.^21^	319	India (South Asia)	An improvement of >1.2 in DAS28 score and a DAS of ≤2.6 (remission) on follow-up	PCR-RFLP	ATIC 347C > G (rs2372536)	The genotype and allele frequencies of ATIC 347C > G SNP did not differ between good and nonresponders and hence this SNP was not found to be associated with MTX treatment response
Ghodke-Puranik Y *et al*.^15^	217	India (South Asia)	ACR50	PCR-RFLP	MTHFR 677C > T (rs1801133); MTHFR 1298A > C (rs1801131); TS (5′UTR repeat and 3′UTR deletion)(rs34489327); MDR1 3435C > T (rs1045642); MDR1 1236C > T (rs1128503); RFC1 80G > A (rs1051266); MS 2756A > G (rs1805087); MTRR 66A > G (rs1801394); GGH −401C > T rs3758149); ATIC 347C > G (rs2372536); SHMT1 1420C > T (rs1979277)	MTHFR 1298A allele (AA-AC) were more likely to have better MTX efficacy relative to those with MTHFR 1298 CC. Similarly, those with an RFC1 80A allele (AA-GA) had better response to MTX than those with the RFC1 80 GG genotyp. None of the other studied SNPs were associated with MTX efficacy in our RA population.
Uribarri M *et al*.^52^	68	Italy (European)	Continued MTX therapy	TaqMan	MTHFR 677C > T (rs1801133)	The results confirm an increased probability of MTX monotherapy discontinuation for RA patients carrying the homozygous 677T variant allele in the MTHFR gene.
Kotrych D *et al*.^41^	422	Poland (European)	DAS28 ≤ 2.2.5 at 6 months of therapy	TaqMan	CXCL9 G > A (rs3733236); CXCL10 G > A (rs8878)	The results of this study suggest lack of associations between the polymorphisms in CXCL9 and CXCL10 genes and the response to MTX in RA patients
Soukup T *et al*.^53^	120	Czech Republic (European)	A mean DAS28 > 2.6 and <3.2 and a reduction in DAS28 > 1.2 during treatment	TaqMan	MTHFR 677C > T (rs1801133), MTHFR 1298A > C (rs1801131)	Data show greater ability of 677CC–1298CC and 677TT–1298AA genotypes to respond to MTX treatment.
Pawlik A *et al*.^37^	156	Poland (European)	DAS28 ≤ 2.4 after 6 months of therapy	Real-Time PCR	ESR1 A > G (rs9340799); ESR1 T > C (rs2234693); ESR2 G > A (rs4986938); ESR2 328G > A (rs1256049)	There were no statistically significant associations of ESR1 and ESR2 gene polymorphisms with response to treatment.
Kurzawski M *et al*.^54^	174	Poland (European)	ACR20	PCR-RFLP	MTHFR 677C > T (rs1801133), MTHFR 1298A > C (rs1801131)	The results of our study suggest that the MTHFR 677T and 1298C alleles may be associated with an increased rate of RA remission in patients treated with MTX receiving high doses of folic acid supplementation.
Wessels JA *et al*.^19^	186	Netherlands (European)	DAS44 ≤ 2.4	Real-Time PCR	MTHFD1 1958G > A (rs17850560); SHMT1 1420C > T (rs17829445); TYMS 28-bp repeat; FPGS 114G > A (rs10760502); FPGS 1994A > G (rs10106); GGH 452C > T (rs11545078); GGH 16T > C (rs1800909)	Only MTHFD1 1958G > A, which compared G allelic carriers with homozygous mutant AA genotypes, showed a possible trend toward a difference between responders and nonresponders
Wessels JA *et al*.^17^	186	Netherlands (European)	DAS44 ≤ 2.4	Real-Time PCR	MTHFR 677C > T (rs1801133), MTHFR 1298A > C (rs1801131)	Patients with MTHFR 1298AA and MTHFR 677CC showed greater clinical improvement with MTX.
Wessels JA *et al*.^20^	205	Netherlands (European)	DAS44 ≤ 2.4	Real-Time PCR	MTRR 66A > G (rs1801394), MTR 2756A > G (rs1805087), AMPD1 34C > T (rs17602729), ITPA 94C > A (rs1127354), and ATIC 347C > G (rs2372536)	AMPD1 34T allele carriers and patients with the ITPA CC genotype and the ATIC 347CC genotype were more likely to achieve good clinical response

DAS28: Disease activity score with 28 joints; DAS44: Disease activity score with 44 joints.

ACR20: American College of Rheumatology 20% response criteria.

ACR50: American College of Rheumatology 50% response criteria.

**Table 2:** Summary of the analyzed studies and the distribution of methylenetetrahydrofolate reductase MTHFR 677 C > T (rs1801133) genotypes.

Study	Study design	Genotype counts						Mean age, years	Mean disease duration, years	MTX dose (mg per week) (range or mean ± s.d.)	Date of end point (week)
		Responders			Nonresponders						
		(Case)			(Control)						
		CC	CT	TT	CC	CT	TT				
Salazar J *et al*.^23^	Prospective cohort	21	17	7	9	6	1	55.62 ± 1.297	5.55	7.5–25	24
Lima A *et al*.^18^	Retrospective cohort	52	46	7	53	53	22	52 ± 11.9	8	15.0 (median) range 2.5–25.0	24
Xiao H *et al*.^48^	Prospective cohort	13	38	11	9	17	5	49.2 ± 13.4	44 ± 13.1	10–15	24
Ghodke Y *et al*.^49^	Retrospective cohort	10	6	1	13	4	0	No information	No information	7.5–17.5	24
Mohammad P. Iqbal *et al*.^50^	Prospective clinical trial	19	7	2	16	5	2	42.87 ± 13.5	Good responder: 6.2 (4.8); Poor responder: 7.0 (4.0)	15–25	24
Ghodke-Puranik Y *et al*.^15^	Retrospective cohort	38	10	1	128	39	1	43.8 ± 10.4	5.6 ± 4.9	15.0 ± 3.9	48
Uribarri M *et al*.^52^	Retrospective cohort	13	28	7	5	8	7	61.5 ± 13.2	13.91 ± 8.11	No information	No information
Soukup T *et al*.^53^	Prospective cohort and retrospective cohort	36	36	8	16	21	3	58.5 ± 12.6	No information	11.7 ± 2.9	24
Kurzawski M *et al*.^54^	Prospective cohort	27	28	3	72	41	3	58.4 ± 11.1	9.7 ± 7.8	7.5–15	24
Wessels JA *et al*.^17^	Prospective cohort	39	39	9	54	55	9	54.6 ± 13.3	<2 years	7.5–15	24

**Table 3:** Associations between gene polymorphisms and MTX efficacy in RA patients.

Outcome	No. of Included studies	Pooled OR	95% CI	Z	P for Z test	I^2^ (%)	Chi-squared	P for Chi-squared
**MTHFR C677T** (**rs1801133**)								
C vs. T (Pre-allele model)								
European	6	1.010	0.731–1.396	0.06	0.951	52	10.43	0.064
East Asian	1	0.823	0.446–1.519	0.62	0.533	—	0.00	—
South Asian	3	0.875	0.522–1.467	0.51	0.612	0	1.31	0.520
Overall	10	0.969	0.768–1.222	0.26	0.792	28	12.50	0.186
CC vs. CT + TT (dominant model)								
European	6	0.947	0.736–1.289	0.18	0.854	16.7	6.01	0.306
East Asian	1	0.649	0.242–1.741	0.86	0.390	—	0.00	—
South Asian	3	0.900	0.505–1.605	0.36	0.721	0	1.12	0.570
Overall	10	0.937	0.734–1.197	0.52	0.604	0	7.75	0.559
TT vs. CC + CT (recessive Model)								
European	6	0.762	0.478–1.215	1.14	0.253	49.9	9.98	0.076
East Asian	1	1.122	0.352–3.570	0.19	0.846	—	0.00	—
South Asian	3	1.580	0.372–6.703	0.62	0.535	0	0.90	0.638
Overall	10	0.851	0.564–1.285	0.77	0.444	24.9	11.99	0.214
CT vs. CC + TT (codominant Model)								
European	6	1.132	0.856–1.498	0.87	0.383	0	4.60	0.467
East Asian	1	1.304	0.545–3.121	-.60	0.551	—	0.00	—
South Asian	3	1.034	0.566–1.889	0.11	0.915	0	0.79	0.672
Overall	10	1.128	0.884–1.439	0.97	0.332	0	5.57	0.782
CC vs. TT (homozygotic Model)								
European	6	1.257	0.766–2.603	0.90	0.366	46.4	9.33	0.097
East Asian	1	0.657	0.169–2.549	0.61	0.543	—	0.00	—
South Asian	3	0.594	0.139–2.541	0.70	0.482	0	0.91	0.635
Overall	10	1.092	0.703–1.696	0.39	0.696	22.7	11.64	0.234
**MTHFR A1298C** (**rs1801131**)								
A vs. C (Pre-allele model)								
European	4	1.021	0.701–1.487	0.11	0.913	48.1	5.78	0.123
East Asian	2	0.694	0.164–2.930	0.50	0.619	76.6	4.28	0.039
South Asian	2	1.201	0.811–1.778	0.91	0.360	0	0.77	0.382
Overall	8	1.004	0.749–1.346	0.03	0.979	43.9	12.49	0.086
AA vs. AC + CC (dominant model)								
European	4	1.135	0.750–1.718	0.60	0.548	20.5	3.77	0.287
East Asian	2	0.658	0.110–3.932	0.46	0.647	73.4	3.76	0.052
South Asian	2	0.671	0.273–1.648	0.87	0.384	21.9	1.28	0.258
Overall	8	0.908	0.596–1.382	0.45	0.652	42.0	12.06	0.099
CC vs. AC + AA (recessive Model)								
European	4	1.238	0.564–2.720	0.53	0.595	42.9	5.25	0.154
East Asian	2	0.824	0.186–3.648	0.26	0.799	0	0.18	0.670
South Asian	2	0.454	0.228–0.906	2.24	0.025	0	0.45	0.504
Overall	8	0.861	0.494–1.503	0.53	0.599	35.9	10.93	0.142
AC vs. AA + CC (codominant Model)								
European	4	0.782	0.551–1.111	1.37	0.170	0	1.09	0.780
East Asian	2	1.707	0.424–6.876	0.75	0.452	61.7	2.61	0.106
South Asian	2	2.319	1.317–4.086	2.91	0.004	0	0.05	0.831
Overall	8	1.205	0.772–1.882	0.82	0.412	55.0	15.54	0.030
AA vs. CC (homozygotic Model)								
European	4	0.819	0.461–1.456	0.68	0.497	52.5	6.31	0.097
East Asian	2	1.296	0.263–6.389	0.32	0.750	0	0.56	0.453
South Asian	2	1.375	0.586–3.224	0.73	0.465	32.5	1.48	0.224
Overall	8	0.987	0.626–1.554	0.06	0.954	24.4	9.25	0.235
**ATIC 347C > G** (**rs2372536**)								
C vs. G (Pre-allele model)								
European	2	1.736	1.176–2.564	2.77	0.006	0	0.13	0.720
East Asian	1	1.503	0.752–3.005	1.15	0.248	—	0.00	—
South Asian	2	0.980	0.730–1.316	0.13	0.895	0	0.01	0.942
Overall	5	1.263	0.958–1.666	1.65	0.098	30.3	5.74	0.220
CC vs. CG + GG (dominant model)								
European	2	2.346	1.407–3.910	3.27	0.001	0	0.20	0.654
East Asian	1	1.474	0.632–3.436	0.90	0.369	—	0.00	—
South Asian	2	1.187	0.734–1.921	0.70	0.485	0	0.53	0.467
Overall	5	1.612	1.168–2.224	2.91	0.004	8.2	4.35	0.360
GG vs. CG + CC (recessive Model)								
European	2	0.770	0.309–1.918	0.56	0.575	0	0.02	0.882
East Asian	1	0.435	0.083–2.279	0.99	0.324	—	0.00	—
South Asian	2	1.277	0.772–2.113	0.95	0.342	0	0.57	0.451
Overall	5	1.068	0.699–1.630	0.30	0.762	0	2.65	0.618
CG vs. CC + GG (codominant Model)								
European	2	0.458	0.274–0.764	2.99	0.003	0	0.09	0.762
East Asian	1	0.824	0.340–1.994	0.43	0.667	—	0.00	—
South Asian	2	0.743	0.489–1.128	1.40	0.163	33.1	1.50	0.221
Overall	5	0.634	0.468–0.858	2.95	0.003	0.9	4.04	0.401
CC vs.GG (homozygotic Model)								
European	2	1.984	0.763–5.161	1.40	0.160	0	0.09	0.764
East Asian	1	2.526	0.468–13.639	1.08	0.281	—	0.00	—
South Asian	2	0.912	0.488–1.704	0.29	0.773	0	0.01	0.941
Overall	5	1.229	0.749–2.015	0.82	0.415	0	2.69	0.611
**TYMS 28**-**bp repeat**								
2R vs. 3R (Pre-allele model)	3	1.174	0.811–1.697	0.85	0.396	55.5	4.49	0.106
2R2R vs. 2R3R + 3R3R (dominant model)	3	1.238	0.794–1.929	0.94	0.347	0	0.75	0.686
3R3R vs. 2R3R + 2R2R (recessive Model)	3	0.787	0.377–1.644	0.64	0.524	72.4	7.24	0.027
2R3R vs. 2R2R + 3R3R(codominant Model)	3	1.093	0.666–1.794	0.35	0.724	50.7	4.06	0.132
2R2R vs. 3R3R (homozygotic Model)	3	1.400	0.675–2.906	0.90	0.366	49.1	3.93	0.14
**MTRR66A > G** (**rs1801394**)								
A vs. G (Pre-allele model)	2	1.088	0.744–1.591	0.43	0.664	0	0.41	0.523
AA vs. AG + GG (dominant model)	2	1.165	0.668–2.031	0.54	0.590	0	0.03	0.864
GG vs. AG + AA (recessive Model)	2	0.961	0.502–1.841	0.12	0.905	0	0.83	0.362
AG vs. AA + GG (codominant Model)	2	0.897	0.531–1.516	0.40	0.686	0	0.07	0.792
AA vs. GG (homozygotic Model)	2	1.188	0.555–2.545	0.44	0.657	0	0.60	0.439
**RFC**-**1 80G > A** (**rs1051266**)								
G vs. A (Pre-allele model)								
European	1	0.561	0.356–0.884	2.49	0.013	—	0.00	—
East Asian	1	1.104	0.615–1.981	0.33	0.740	—	0.00	—
South Asian	2	0.705	0.523–0.951	2.29	0.022	5.9	1.06	0.303
Overall	4	0.716	0.545–0.941	2.39	0.017	30.0	4.29	0.232
GG vs. GA + AA (dominant model)								
European	1	0.480	0.204–1.130	1.68	0.093	—	0.00	—
East Asian	1	1.086	0.376–3.135	0.15	0.878	—	0.00	—
South Asian	2	0.642	0.415–0.993	1.99	0.046	34.7	1.53	0.216
Overall	4	0.645	0.449–0.926	2.38	0.017	0	2.92	0.405
AA vs. GA + GG (recessive Model)								
European	1	2.343	1.169–4.694	2.40	0.016	—	0.00	—
East Asian	1	0.824	0.322–2.109	0.40	0.687	—	0.00	—
South Asian	2	1.705	0.980–2.964	1.89	0.059	0	0	0.952
Overall	4	1.653	1.115–2.451	2.50	0.012	2.9	3.09	0.378
GA vs. GG + AA (codominant Model)								
European	1	0.785	0.418–1.476	0.75	0.453	—	0.00	—
East Asian	1	1.102	0.481–2.525	0.23	0.818	—	0.00	—
South Asian	2	1.113	0.742–1.668	0.52	0.606	13.2	1.15	0.283
Overall	4	1.018	0.743–1.396	0.11	0.910	0	2.02	0.567
GG vs. AA (homozygotic Model)								
European	1	0.301	0.114–0.796	2.42	0.016	—	0.00	—
East Asian	1	1.235	0.353–4.320	0.33	0.741	—	0.00	—
South Asian	2	0.473	0.252–0.887	2.33	0.020	0	0.33	0.563
Overall	4	0.488	0.302–0.789	2.93	0.003	12.1	3.41	0.332
**SLC19A1 G** > **A** (**rs7499**)								
G vs. A (Pre-allele model)	2	1.536	1.176–2.005	3.15	0.002	0	0.02	0.893
GG vs. GA + AA (dominant model)	2	1.681	1.146–2.467	2.66	0.008	0	0.19	0.66
AA vs. GA + GG (recessive Model)	2	0.528	0.316–0.884	2.43	0.015	0	0.49	0.484
GA vs. GG + AA (codominant Model)	2	0.861	0.596–1.242	0.80	0.423	0	0.73	0.394
GG vs. AA (homozygotic Model)	2	2.397	1.359–4.229	3.02	0.003	0	0.10	0.753
**SLC19A1 A > G** (**rs2838956**)								
A vs. G (Pre-allele model)	2	1.366	1.051–1.776	2.33	0.020	0	0.21	0.644
AA vs. AG + GG (dominant model)	2	1.426	0.965–2.109	1.78	0.075	8.2	1.09	0.297
GG vs. AG + AA (recessive Model)	2	0.592	0.361–0.969	2.08	0.037	0	0.09	0.764
AG vs. AA + GG (codominant Model)	2	0.980	0.635–1.512	0.09	0.927	28.6	1.40	0.237
AA vs. GG (homozygotic Model)	2	1.973	1.131–3.443	2.39	0.017	0	0.10	0.754
**ABCB1 C3435T** (**rs1045642**)								
C vs. T (Pre-allele model)	2	1.714	0.65–4.522	1.09	0.276	86.9	7.63	0.006
CC vs. CT + TT (dominant model)	2	1.755	0.573–5.372	0.99	0.325	75.4	4.07	0.044
TT vs. CC + CT (recessive Model)	2	0.429	0.093–1.985	1.08	0.279	83.4	6.04	0.014
CT vs. CC + TT (codominant Model)	2	1.033	0.666–1.602	0.15	0.884	0	0.04	0.838
CC vs. TT (homozygotic Model)	2	2.973	0.401–22.016	1.07	0.286	86.6	7.48	0.006

**Table 4:** Summary of the analyzed studies and the distribution of methylenetetrahydrofolate reductase MTHFR 1298A > C (rs1801131) genotypes.

Study	Study design	Genotype counts						Mean age, years	Mean disease duration, years	MTX dose (mg per week) (range or mean ± s.d.)	Date of end point (week)
		Responders			Nonresponders						
		(Case)			(Control)						
		AA	AC	CC	AA	AC	CC				
Salazar J *et al*.^23^	Prospective cohort	24	16	5	6	6	4	55.62 ± 1.297	5.55	7.5–25	24
Xiao H *et al*.^48^	Prospective cohort	37	24	1	26	5	0	49.2 ± 13.4	44 ± 13.1	10–15	24
Ghodke Y *et al*.^49^	Retrospective cohort	6	8	3	4	9	4	No information	No information	7.5–17.5	24
Mohammad P. Iqbal *et al*.^50^	Prospective clinical trial	2	19	6	5	11	7	42.87 ± 13.5	Good responder: 6.2 (4.8); Poor responder: 7.0 (4.0)	15–25	24
Ghodke-Puranik Y *et al*.^15^	Retrospective cohort	12	29	8	46	66	56	43.8 ± 10.4	5.6 ± 4.9	15.0 ± 3.9	48
Soukup T *et al*.^53^	Prospective cohort and retrospective cohort	38	33	9	19	20	1	58.5 ± 12.6	No information	11.7 ± 2.9	24
Kurzawski M *et al*.^54^	Prospective cohort	16	31	11	41	62	13	58.4 ± 11.1	9.7 ± 7.8	7.5–15	24
Wessels JA *et al*.^17^	Prospective cohort	41	36	10	43	60	13	54.6	<2 years	7.5–15	24

**Table 5:** Summary of the analyzed studies and the distribution of methylenetetrahydrofolate reductase ATIC 347C > G (rs2372536) genotypes.

Study	Study design	Genotype counts						Mean age, years	Mean disease duration, years	MTX dose (mg per week) (range or mean ± s.d.)	Date of end point (week)
		Responders			Nonresponders						
		(Case)			(Control)						
		CC	CG	GG	CC	CG	GG				
Ghodke-Puranik Y *et al*.^15^	Retrospective cohort	11	25	13	38	83	42	43.8 ± 10.4	5.6 ± 4.9	15.0 ± 3.9	48
Takatori R *et al*.^35^	Prospective cohort	48	21	3	19	11	3	59.2	4.04	6	>20
Salazar J *et al*.^23^	Prospective cohort	26	15	4	5	9	2	55.62 ± 1.297	5.55	7.5–25	>24
Wessels JA *et al*.^20^	Prospective cohort	51	30	6	46	62	10	54.6 ± 13.3	<2 years	15 or 25	>18
Sharma S *et al*.^22^	Prospective cohort	61	97	47	16	41	11	GR* 45.0 ± 11.50, PR 40.9 ± 12.7	<5 years	Up to 25	>24

*GR: Good response; PR: Poor response.

**Table 6:** Summary of the analyzed studies and the distribution of methylenetetrahydrofolate reductase TYMS 28 bp VNTR (rs34743033) genotypes.

Study	Study design	Genotype counts						Mean age, years	Mean disease duration, years	MTX dose (mg per week) (range or mean ± s.d.)	Date of end point (week)
		Responders			Nonresponders						
		(Case)			(Control)						
		2R2R	2R3R	3R3R*	2R2R	2R3R	3R3R*				
Wessels JA *et al*.^19^	Retrospective cohort	19	39	29	20	53	26	Responder: 55.3 ± 14;Nonresponder: 53.6 ± 13	<2 years	7.5–15	24
Lima A *et al*.^18^	Retrospective cohort	19	62	21	16	60	48	52 ± 11.9	Median disease duration: 8	15.0 (median) range 2.5–25.0	MTX median disease duration: 28 months
Jekic B *et al*.^25^	Prospective cohort	27	82	37	7	20	11	58.04 ± 10.20	48.95 ± 39.95 months	10.72 ± 2.83	24

*3R4R genotype (n = 4) was excluded from the analyses because of the low frequency.

**Table 7:** Summary of the analyzed studies and the distribution of methylenetetrahydrofolate reductase MTRR 66A > G (rs1801394) genotypes.

Study	Study design	Genotype counts						Mean age, years	Mean disease duration, years	MTX dose (mg per week) (range or mean ± s.d.)	Date of end point (week)
		Responders			Nonresponders						
		(Case)			(Control)						
		AA	AG	GG	AA	AG	GG				
Shen S.-H *et al*.^26^	Prospective cohort	50	23	4	18	9	3	50.1 ± 13.8	3.8 ± 2.4	10–15	24
Ghodke-Puranik Y *et al*.^15^	Retrospective cohort	13	22	14	41	82	45	43.8 ± 10.4	5.6 ± 4.9	15.0 ± 3.9	48

**Table 8:** Summary of the analyzed studies and the distribution of methylenetetrahydrofolate reductase RFC-1 80G > A (rs1051266) genotypes.

Study	Study design	Genotype counts						Mean age, years	Mean disease duration, years	MTX dose (mg per week) (range or mean ± s.d.)	Date of end point (week)
		Responders			Nonresponders						
		(Case)			(Control)						
		GG	GA	AA	GG	GA	AA				
Muralidharan N *et al*.^27^	Prospective cohort	34	64	21	35	59	12	42.73 ± 0.56	3.76 ± 0.23	16.75 ± 4	16
Takatori R *et al*.^35^	Prospective cohort	14	41	17	6	18	9	59.2	4.04	6	8
Ghodke-Puranik Y *et al*.^15^	Retrospective cohort	13	25	11	74	70	24	43.8 ± 10.4	5.6 ± 4.9	15.0 ± 3.9	48
Drozdzik M *et al*.^27^	Prospective cohort	8	28	22	29	63	24	21–70	7.9	7.5–15	24

**Table 9:** Summary of the analyzed studies and the distribution of methylenetetrahydrofolate reductase SLC19A1 G > A (rs7499) genotypes.

Study	Study design	Genotype counts						Mean age, years	Mean disease duration, years	MTX dose (mg per week) (range or mean ± s.d.)	Date of end point (week)
		Responders			Nonresponders						
		(Case)			(Control)						
		GG	GA	AA	GG	GA	AA				
Lima A *et al*.^34^	Retrospective cohort	47	47	11	44	57	27	52 ± 11.9	Median disease duration: 8	15.0 (median) range 2.5–25.0	MTX median disease duration: 28 months
Owen SA *et al*.^46^	Retrospective cohort	59	64	18	27	51	18	Median age: 54.2	Responders: 7.3 (6.8–8.4); Inefficacy: 6.4 (5.8–10.4)	>15	24

**Table 10:** Summary of the analyzed studies and the distribution of methylenetetrahydrofolate reductase SLC19A1 A > G (rs2838956) genotypes.

Study	Study design	Genotype counts						Mean age, years	Mean disease duration, years	MTX dose (mg per week) (range or mean ± s.d.)	Date of end point (week)
		Responders			Nonresponders						
		(Case)			(Control)						
		AA	AG	GG	AA	AG	GG				
Lima A *et al*.^34^	Retrospective cohort	39	52	14	43	57	28	52 ± 11.9	Median disease duration: 8	15.0 (median) range 2.5–25.0	MTX median disease duration: 28 months
Owen SA *et al*.^46^	Retrospective cohort	52	70	19	24	54	19	Median age: 54.2	Responders: 7.3 (6.8–8.4); Inefficacy: 6.4 (5.8–10.4)	>15	24

**Table 11:** Summary of the analyzed studies and the distribution of methylenetetrahydrofolate reductase ABCB1 3435C > T (rs1045642) genotypes.

Study	Study design	Genotype counts						Mean age, years	Mean disease duration, years	MTX dose (mg per week) (range or mean ± s.d.)	Date of end point (week)
		Responders			Nonresponders						
		(Case)			(Control)						
		CC	CT	TT	CC	CT	TT				
Takatori R *et al*.^35^	Prospective cohort	34	32	6	7	15	11	59.2	4.04	6	8
Lima A *et al*.^34^	Retrospective cohort	29	50	26	34	59	35	52 ± 11.9	Median disease duration: 8	15.0 (median) range 2.5–25.0	MTX median disease duration: 28 months

## References

[b1] Kung T. N. . RFC1 80G>A is a genetic determinant of methotrexate efficacy in rheumatoid arthritis: a human genome epidemiologic review and meta-analysis of observational studies. Arthritis Rheumatol 66, 1111–1120 (2014). 24782176 10.1002/art.38331

[b2] Li X. . The association between reduced folate carrier-1 gene 80G/A polymorphism and methotrexate efficacy or methotrexate related-toxicity in rheumatoid arthritis: A meta-analysis. Int Immunopharmacol 38, 8–15 (2016). 27233001 10.1016/j.intimp.2016.05.012

[b3] Owen S. A. . MTHFR gene polymorphisms and outcome of methotrexate treatment in patients with rheumatoid arthritis: analysis of key polymorphisms and meta-analysis of C677T and A1298C polymorphisms. Pharmacogenomics J 13, 137–147 (2013). 21931346 10.1038/tpj.2011.42

[b4] Song G. G., Bae S. C. & Lee Y. H. Association of the MTHFR C677T and A1298C polymorphisms with methotrexate toxicity in rheumatoid arthritis: a meta-analysis. Clin Rheumatol 33, 1715–1724 (2014). 24794492 10.1007/s10067-014-2645-8

[b5] Y. H. Lee S.-C. B. & Song G. G. Association of the ABCB1 C3435T polymorphism with responsiveness to and toxicity of DMARDs in rheumatoid arthritis A meta-analysis. Z Rheumatol, doi: 10.1007/s00393-00015-1618-x (2015). 26184955

[b6] Kyburz D., Gabay C., Michel B. A. & Finckh A. The long-term impact of early treatment of rheumatoid arthritis on radiographic progression: a population-based cohort study. Rheumatology (Oxford) 50, 1106–1110 (2011). 21258051 10.1093/rheumatology/keq424

[b7] Finckh A., Liang M. H., van Herckenrode C. M. & de Pablo P. Long-term impact of early treatment on radiographic progression in rheumatoid arthritis: A meta-analysis. Arthritis Rheum 55, 864–872 (2006). 17139662 10.1002/art.22353

[b8] Romão V. C., Lima A., Bernardes M., Canhão H. & Fonseca J. E. Three decades of low-dose methotrexate in rheumatoid arthritis: can we predict toxicity. Immunol Res 60, 289–310 (2014). 25391609 10.1007/s12026-014-8564-6

[b9] Malik F. & Ranganathan P. Methotrexate pharmacogenetics in rheumatoid arthritis: a status report. Pharmacogenomics 14, 305–314 (2013). 23394392 10.2217/pgs.12.214

[b10] Verstappen S. M. . Adverse events and factors associated with toxicity in patients with early rheumatoid arthritis treated with methotrexate tight control therapy: the CAMERA study. Ann Rheum Dis 69, 1044–1048 (2010). 19581281 10.1136/ard.2008.106617

[b11] Zhu H., Deng F. Y., Mo X. B., Qiu Y. H. & Lei S. F. Pharmacogenetics and pharmacogenomics for rheumatoid arthritis responsiveness to methotrexate treatment: the 2013 update. Pharmacogenomics 15, 551–566 (2014). 24624921 10.2217/pgs.14.25

[b12] Lee Y. H. & Song G. G. Associations between the C677T and A1298C polymorphisms of MTHFR and the efficacy and toxicity of methotrexate in rheumatoid arthritis: a meta-analysis. Clin Drug Investig 30, 101–108 (2010). 10.2165/11531070-000000000-00000 20067328

[b13] Morgan M. D. . MTHFR functional genetic variation and methotrexate treatment response in rheumatoid arthritis: a meta-analysis. Pharmacogenomics 15, 467–475 (2014). 24624914 10.2217/pgs.13.235

[b14] Lee Y. H. & Bae S. C. Association of the ATIC 347 C/G polymorphism with responsiveness to and toxicity of methotrexate in rheumatoid arthritis: a meta-analysis. Rheumatol Int, doi: 10.1007/s00296-00016-3523-2 (2016). 27379764

[b15] Ghodke-Puranik Y. . Folate metabolic pathway single nucleotide polymorphisms: a predictive pharmacogenetic marker of methotrexate response in Indian (Asian) patients with rheumatoid arthritis. Pharmacogenomics 16, 2019–2034 (2015). 26616421 10.2217/pgs.15.145 PMC4976849

[b16] Ranganathan P. An update on methotrexate pharmacogenetics in rheumatoid arthritis. Pharmacogenomics 9, 439–451 (2008). 18384257 10.2217/14622416.9.4.439

[b17] Wessels J. A. . Efficacy and toxicity of methotrexate in early rheumatoid arthritis are associated with single-nucleotide polymorphisms in genes coding for folate pathway enzymes. Arthritis Rheum 54, 1087–1095 (2006). 16572443 10.1002/art.21726

[b18] Aurea Lima J. M., Miguel Bernardes H. S., Rita Azevedo V. S. & Medeiros R. Prediction of Methotrexate Clinical Response in Portuguese Rheumatoid Arthritis Patients: Implication of MTHFR rs1801133 and ATIC rs4673993 Polymorphisms. Hindawi Publishing Corporation BioMed Research International 2014, Article ID 368681, 11 pages (2014). 10.1155/2014/368681 PMC4055378 24967362

[b19] Wessels J. A. . A clinical pharmacogenetic model to predict the efficacy of methotrexate monotherapy in recent-onset rheumatoid arthritis. Arthritis Rheum 56, 1765–1775 (2007). 17530705 10.1002/art.22640

[b20] Judith A. M., Wessels W. M. K. . Relationship Between Genetic Variants in the Adenosine Pathway and Outcome of Methotrexate Treatment in Patients With Recent-Onset Rheumatoid Arthritis. Arthritis & rheumatism 54, 2830–2839 (2006). 16947783 10.1002/art.22032

[b21] Muralidharan N., Mariaselvam C. M., Jain V. K., Gulati R. & Negi V. S. ATIC 347C>G gene polymorphism may be associated with methotrexate-induced adverse events in south Indian Tamil rheumatoid arthritis. Pharmacogenomics 17, 241–248 (2016). 26799664 10.2217/pgs.15.170

[b22] Shruti Sharma M. D. . Purine biosynthetic pathway genes and methotrexate response in rheumatoid arthritis patients among north Indians. Pharmacogenet Genomics 19, 823–828 (2009). 19902562 10.1097/fpc.0b013e328331b53e

[b23] Salazar J. . Polymorphisms in genes involved in the mechanism of action of methotrexate: are they associated with outcome in rheumatoid arthritis patients. Pharmacogenomics 15, 1079–1090 (2014). 25084201 10.2217/pgs.14.67

[b24] Lima A. . Role of key TYMS polymorphisms on methotrexate therapeutic outcome in portuguese rheumatoid arthritis patients. PLoS One 9, e108165 (2014). 25279663 10.1371/journal.pone.0108165 PMC4184792

[b25] Jekic B. . Association of the TYMS 3G/3G genotype with poor response and GGH 354GG genotype with the bone marrow toxicity of the methotrexate in RA patients. Eur J Clin Pharmacol 69, 377–383 (2013). 22763757 10.1007/s00228-012-1341-3

[b26] SHEN Shi-hua X. U. J., XU Sheng-qian L. L. & L. I. Ying-wei X. H. The relationship between the single nucleotide polymorphism of MTR, MTRR gene and the treatmentof methotrexate in rheumatoid arthriti. Chinese Pharmacological Bulletin 25, 1068–1071 (2009).

[b27] Muralidharan N., Mariaselvam C. M., Cb M. & Negi V. S. Reduced folate carrier-1 80G>A gene polymorphism is not associated with methotrexate treatment response in South Indian Tamils with rheumatoid arthritis. Clin Rheumatol 35, 879–885 (2016). 25771854 10.1007/s10067-015-2917-y

[b28] Bohanec G. P., Logar D., Lestan B. & Dolzan V. Genetic determinants of methotrexate toxicity in rheumatoid arthritis patients: a study of polymorphisms affecting methotrexate transport and folate metabolism. Eur J Clin Pharmacol 64, 1057–1068 (2008). 18607581 10.1007/s00228-008-0521-7

[b29] Dervieux T. . Gene-gene interactions in folate and adenosine biosynthesis pathways affect methotrexate efficacy and tolerability in rheumatoid arthritis. Pharmacogenet Genomics 19, 935–944 (2009). 19858780 10.1097/FPC.0b013e32833315d1

[b30] Hayashi H. . A single nucleotide polymorphism of reduced folate carrier 1 predicts methotrexate efficacy in Japanese patients with rheumatoid arthritis. Drug Metab Pharmacokinet 28, 164–168 (2013). 22971639 10.2133/dmpk.dmpk-12-nt-038

[b31] James H. M. . Common polymorphisms in the folate pathway predict efficacy of combination regimens containing methotrexate and sulfasalazine in early rheumatoid arthritis. J Rheumatol 35, 562–571 (2008). 18322994

[b32] Chatzikyriakidou A. . Transcription regulatory polymorphism −43T>C in the 5′-flanking region of SLC19A1 gene could affect rheumatoid arthritis patient response to methotrexate therapy. Rheumatol Int 27, 1057–1061 (2007). 17404734 10.1007/s00296-007-0339-0

[b33] Stamp L. K. & Roberts R. L. Effect of genetic polymorphisms in the folate pathway on methotrexate therapy in rheumatic diseases. Pharmacogenomics 12, 1449–1463 (2011). 22008049 10.2217/pgs.11.86

[b34] Aurea Lima M. B., Rita Azevedo R. M. & Seabra V. Pharmacogenomics of Methotrexate Membrane Transport Pathway: Can Clinical Response to Methotrexate in Rheumatoid Arthritis Be Predicted. Int J Mol Sci 16, 13760–13780 (2015). 26086825 10.3390/ijms160613760 PMC4490522

[b35] Takatori R. . ABCB1 C3435T polymorphism influences methotrexate sensitivity in rheumatoid arthritis patients. Clin Exp Rheumatol 24, 546–554 (2006). 17181924

[b36] Pawlik A. . Effect of allograft inflammatory factor-1 gene polymorphisms on rheumatoid arthritis treatment with methotrexate. Postepy Hig Med Dosw (Online) 67, 637–642 (2013). 24018427 10.5604/17322693.1058897

[b37] Andrzej Pawlik V. D., Mateusz Kurzawski K. S. & Daniel Kotrych A. B. Effect of ESR1 and ESR2 gene polymorphisms on rheumatoid arthritis treatment with methotrexate. Pharmacol Rep 64 (2012). 10.1016/s1734-1140(12)70745-4 22580535

[b38] Edyta Majorczyk A. P. & Kuśnierczyk P. PTPN22 1858CNT polymorphism is strongly associated with rheumatoid arthritis but not with a response to methotrexate therapy. Int Immunopharmacol 2010, 1626–1629 (2010). 10.1016/j.intimp.2010.09.008 20888443

[b39] Azra Arif Ali T. M., Jawed Altaf B., Ahmed Iqbal A. H. & Iqbal M. P. Polymorphism of HLA-DR and HLA-DQ in rheumatoid arthritis patients and clinical response to methotrexate - a hospital-based study. J Pak Med Assoc 56, 452–456 (2006). 17144392

[b40] Kooloos W. M. . Functional polymorphisms and methotrexate treatment outcome in recent-onset rheumatoid arthritis. Pharmacogenomics 11, 163–175 (2010). 20136356 10.2217/pgs.09.139

[b41] Kotrych D., Dziedziejko V., Safranow K. & Pawlik A. Lack of association between CXCL9 and CXCL10 gene polymorphisms and the outcome of rheumatoid arthritis treatment with methotrexate. Eur Rev Med Pharmacol Sci 19, 3037–3040 (2015). 26367725

[b42] Moher D., Liberati A., Tetzlaff J. & Altman D. G. Preferred reporting items for systematic reviews and meta-analyses: the PRISMA statement. BMJ 339, b2535 (2009). 19622551 10.1136/bmj.b2535 PMC2714657

[b43] Fisher M. C. & Cronstein B. N. Metaanalysis of methylenetetrahydrofolate reductase (MTHFR) polymorphisms affecting methotrexate toxicity. J Rheumatol 36, 539–545 (2009). 19208607 10.3899/jrheum.080576 PMC2673494

[b44] Świerkot J. . Associations between single-nucleotide polymorphisms of RFC-1, GGH, MTHFR, TYMS, and TCII genes and the efficacy and toxicity of methotrexate treatment in patients with rheumatoid arthritis. Pol Arch Med Wewn 125, 152–161 (2015). 25599563 10.20452/pamw.2707

[b45] Fanfan Zheng Y. Z. . Further evidence for genetic association of CACNA1C and schizophrenia: New risk loci in a Han Chinese population and a meta-analysis. Schizophr Res 152, 105–110 (2014). 24355530 10.1016/j.schres.2013.12.003

[b46] Owen S. A. . Genetic polymorphisms in key methotrexate pathway genes are associated with response to treatment in rheumatoid arthritis patients. Pharmacogenomics J 13, 227–234 (2013). 22450926 10.1038/tpj.2012.7 PMC5604731

[b47] Milic V. . Association of dihydrofolate reductase (DHFR) -317AA genotype with poor response to methotrexate in patients with rheumatoid arthritis. Clin Exp Rheumatol 30, 178–183 (2012). 22324981

[b48] Xiao H. . Associations between the genetic polymorphisms of MTHFR and outcomes of methotrexate treatment in rheumatoid arthritis. Clin Exp Rheumatol 28, 728–733 (2010). 20863444

[b49] Ghodke Y., Chopra A., Joshi K. & Patwardhan B. Are Thymidylate synthase and Methylene tetrahydrofolate reductase genes linked with methotrexate response (efficacy, toxicity) in Indian (Asian) rheumatoid arthritis patients. Clin Rheumatol 27, 787–789 (2008). 18274813 10.1007/s10067-008-0852-x

[b50] Iqbal M. P., Ali A. A., Mehboobali N. & Iqbal K. Short Communication: Lack of association between MTHFR gene polymorphisms and response to methotrexate treatment in Pakistani patients with rheumatoid arthritis. Pak J Pharm Sci 28, 1789–1792 (2015). 26408898

[b51] Aggarwal P., Naik S., Mishra K. P., Aggarwal A. & Misra R. Correlation between methotrexate efficacy & toxicity with C677T polymorphism of the methylenetetrahydrofolate gene in rheumatoid arthritis patients on folate supplementation. Indian J Med Res 124, 521–526 (2006). 17213520

[b52] Uribarri M. . Influence of MTHFR C677T polymorphism on methotrexate monotherapy discontinuation in rheumatoid arthritis patients: results from the GAPAID European project. Clin Exp Rheumatol 33, 699–705 (2015). 26314492

[b53] Soukup T. . The impact of C677T and A1298C MTHFR polymorphisms on methotrexate therapeutic response in East Bohemian region rheumatoid arthritis patients. Rheumatol Int 35, 1149–1161 (2015). 25618758 10.1007/s00296-015-3219-z

[b54] Kurzawski M., Pawlik A., Safranow K., Herczynska M. & Drozdzik M. 677C>T and 1298A>C MTHFR polymorphisms affect methotrexate treatment outcome in rheumatoid arthritis. Pharmacogenomics 8 , 1551–1559 (2007). 18034620 10.2217/14622416.8.11.1551

